# Learning-based sliding mode synchronization for fractional-order Hindmarsh-Rose neuronal models with deterministic learning

**DOI:** 10.3389/fnins.2023.1246778

**Published:** 2023-09-27

**Authors:** Danfeng Chen, Junsheng Li, Chengzhi Yuan, Jun He, Wenbo Zhu

**Affiliations:** ^1^School of Mechatronic Engineering and Automation, Foshan University, Foshan, China; ^2^Department of Mechanical, Industrial and Systems Engineering, University of Rhode Island, Kingston, RI, United States

**Keywords:** fractional-order Hindmarsh-Rose system, synchronization control, sliding mode control, deterministic learning, system identification

## Abstract

**Introduction:**

In recent years, extensive research has been conducted on the synchronous behavior of neural networks. It is found that the synchronization ability of neurons is related to the performance of signal reception and transmission between neurons, which in turn affects the function of the organism. However, most of the existing synchronization methods are faced with two difficulties, one is the structural parameter dependency, which limits the promotion and application of synchronous methods in practical problems. The other is the limited adaptability, that is, even when faced with the same control tasks, for most of the existing control methods, the control parameters still need to be retrained. To this end, the present study investigates the synchronization problem of the fractional-order HindmarshRose (FOHR) neuronal models in unknown dynamic environment.

**Methods:**

Inspired by the human experience of knowledge acquiring, memorizing, and application, a learning-based sliding mode control algorithm is proposed by using the deterministic learning (DL) mechanism. Firstly, the unknown dynamics of the FOHR system under unknown dynamic environment is locally accurately identified and stored in the form of constant weight neural networks through deterministic learning without dependency of the system parameters. Then, based on the identified and stored system dynamics, the model-based and relearning-based sliding mode controller are designed for similar as well as new synchronization tasks, respectively.

**Results:**

The synchronization process can be started quickly by recalling the empirical dynamics of neurons. Therefore, fast synchronization effect is achieved by reducing the online computing time. In addition, because of the convergence of the identification and synchronization process, the control experience can be constantly replenished and stored for reutilization, so as to improve the synchronization speed and accuracy continuously.

**Discussion:**

The thought of this article will also bring inspiration to the related research in other fields.

## 1. Introduction

In recent years, the dynamic behavior of complex networks, especially neural networks, has attracted extensive attention. It is found that the performance of signal reception and transmission between neurons affecting the functions of the organism depends on the synchronization ability of neurons. The most commonly mentioned neurological diseases such as Epileptic, Parkinson's, Alzheimer's, autism, and schizophrenia, are closely related to the synchronization ability of brain neurons (Yang et al., 2021; Zeng et al., 2023). Precisely, it has been proved that decreased synchronization can lead to neural disorders such as schizophrenia, while increased synchronization abnormalities may induce neurological diseases such Parkinson's disease and epilepsy (Uhlhaas et al., 2006). In addition, as presented in Brown et al. (2004), the firing rate of neurons in the subthalamic basement nucleus (STN) and the medial Globus Pallidus (GPI) in Parkinson's patients not only increased but also tended to synchronize abnormally.

For these neuropathies mentioned above, electrical stimulation method (Liu et al., 2019) was the most commonly used clinical treatment method. The abnormal synchronization of neurons is calibrated by adjusting the direction, frequency, and amplitude of the stimulation current. However, for different neurological diseases, how to choose or set optimal parameters of the stimulation current is a difficult problem.

Considering that the process of information generation, transmission, and decoding between neurons are closely related to their complex discharge activities, it is of paramount significance to simulate the electrical activity of neurons through mathematical models. Thus, many research studies are conducted on differential equation models to further analyze the influence of the parameter variations on the neuronal electrophysiological processes and firing activities. Among the various differential neuron models, such as the Hodgin-Huxley (HH) (Hodgkin and Huxley, 1952), FitzHugh-Nagumo (FHN) (Fitzhugh, 1961), Hindmarsh-Rose (HR) (Hindmarsh et al., 1984) and Ermentrout (Ermentrout, 2014) neuronal models, the HR neuronal model is the most commonly used one for non-linear dynamic and synchronization analysis (Parastesh et al., 2019; Liu et al., 2021; Remi et al., 2022).

The HR model possesses simple polynomial expression and can accurately describe the process of signal transmission across neurons. In Boaretto et al. (2018), the HR model was introduced to study the dynamic mechanism of abnormal phase synchronization. As discussed in Simo et al. (2021), the effect of the electromagnetic on the HR model under weak electric environment was considered to simulate the electrical activities and the synchronization process of neurons. In Ding et al. (2022), the dynamics of fractional-order memristor-coupled Hindmarsh-Rose neuron model considering synaptic crosstalk was investigated. It revealed that there were differences between the number and stability of equilibrium points for different crosstalk strength parameters. As discussed in Li et al. (2023), Fourier coefficients are introduced to investigate the effect of electric field on vibrational resonance for signal detection in a single neuron model and a bidirectionally coupled neuron model, respectively. It was found that the periodic external electric field of an appropriate frequency significantly enhances the vibrational resonance, which indicate that the external electric field may play a constructive role in the detection of weak signals in the brain and neuronal systems. In addition, the Hopf bifurcation, one of the typical non-linear dynamic behaviors was investigated in a memristor-coupled Hindmarsh-Rose and FitzHugh-Nagumo neurons with two time delays in Guo et al. (2023). It revealed that the time delay in HR neurons has a greater effect on blocking the synchronization than the time delay in FHN neuron.

Apart from the dynamic analysis using differential models, a large number of electrophysiological experiments have been conducted for the electrophysiological mechanism of abnormal synchronization of neurons (Jia et al., 2012). Through biological experiments given by Gu et al. (2014), it was found that the discharge frequency of the neuronal system became faster with the increase of potassium (*K*^+^) concentration. Furthermore, with the variation of the concentration of potassium, the neuronal system showed different firing models, which was consistent with the dynamic performance of HR model under external stimulus current. In Jia et al. (2017), the authors conducted corresponding biological experiments on the calcium ion (*Ca*^2+^) of neuron cells. Similar results have been achieved, that is, with the change of calcium concentration in a certain range, the neurons exhibit single-cycle, double-cycle, and chaotic electrical activity. In , it indicates that by adjusting the concentration of calcium ions (*Ca*^2+^) of neurons, the corresponding inter spike interval (ISI) demonstrates similar features with the dynamic behaviors of the HR model with the variation of system parameters. These results further demonstrate that it is feasible and effective to analyze the electrical activity of neurons according to the non-linear dynamics of the HR model with different system parameters, such as the external stimulating current and other related parameters.

Recently, increasing attention has been focused on fractional-order (FO) calculus (Rihan et al., 2019; Wang et al., 2020; Jin et al., 2021), which is also very popular in the field of neuroscience (Dong et al., 2014). It was found that compared with the integer order model, the fractional neuron models reveal more advantages, such as the FO neuron models can describe the physical memory and genetics more accurately and can illustrate the biological characteristics more correctly in the presence of noise (Dong et al., 2014). Moreover, the stimulating dynamical features show that many neural computing features can be implemented in FO systems, which enriches the functional neuronal mechanisms. Therefore, the neural dynamic analysis method based on fractional HR model makes the model-based modeling of abnormal synchronization of neurons step up to a new stage.

In addition to the mechanism analysis based on the model and biological experiments, the synchronization control between neurons is also one of the core problems attracting people's attention. Over the past few decades, various control techniques, including neural network control (Motallebzadeh et al., 2012), feedback control (Semenov and Fradkov, 2021), adaptive control (Deng et al., 2006), fuzzy control (Nirvin et al., 2021), and sliding mode control (Chen et al., 2012; Vafaei et al., 2019), have been proposed and applied to the control and synchronization of the HR model as well as the FOHR models. As presented in Rajagopal et al. (2019), a feedback synchronization controller was designed for the fractional-order HR neuronal model, whose gain was limited to some parameter conditions. The authors in Giresse et al. (2019) designed controllers for the synchronized behaviors of coupled FOEHR neurons. Among these methods, sliding mode control and adaptive control techniques have attracted much attention due to their positive features such as guaranteed stability, strong robustness against parameter variations, and simplicity in implementation (Meng et al., 2020). As presented in Che et al. (2010), for unidirectional complete synchronization of HR neurons, a sliding mode control scheme with additional conditions was considered. However, the chattering phenomena is the main problem faced by the sliding mode control methods. Thus, many research studies are conducted to reduce the chattering problem by using different sliding mode surfaces. However, most of the results show that there is a tradeoff between control error and the control smoothness. In addition to the problem mentioned above, most of these control methods depend heavily on the system models. For most of the actual dynamic systems, the system models have some uncertainty because of the influence of dynamic environment (Rabah et al., 2017; Xu et al., 2020). How to avoid the influence of system uncertainty and disturbance on the control system performance is of great significance for the synchronization control of chaotic system. As discussed in Liu et al. (2021), the adaptive radial basis function (RBF) neural network was introduced for the identification of the unknown system dynamics of the HR model. However, the training time and computation cost of neural network observer inevitably increase greatly.

In Wang and Hill (2018), Wang proposed a deterministic learning (DL) theory mainly discussing the problem of knowledge learning and reutilization of non-linear dynamic systems under unknown dynamic environment by using the RBF neural networks. It has proved that for any period or period-like system input, the persistence of excitation (PE) condition can be satisfied and the precise convergence of the neural network weights can be achieved. With the development of deterministic learning theory, it was further applied for the problem of dynamic pattern recognition (Lin et al., 2019), period-doubling bifurcation detection (Chen and Wang, 2016), and intelligent control (Zhang et al., 2023). The DL algorithm emphasizes the preservation and reutilization of system dynamic knowledge. When faced with similar recognition or control tasks, it can quickly recall the identified and stored system knowledge so as to reduce the online computation time.

Inspired by the above discussion, the dynamic characteristics of the HR model, especially the FOHR neuron model under unknown dynamic environment is considered in this study and the model-based and learning-based sliding mode control algorithm are proposed by using the deterministic learning (DL) mechanism. Since the system dynamics of the slave system is unknown as considered in this study, in order to achieve ideal robustness effect of the control system, the traditional sliding mode control method usually sets too large gain parameters to overcome the system uncertainties, which in turn leads to serious chattering problem. In our study, the sliding mode gain parameter is effectively reduced by compensating the system dynamics with locally accurate system identification. First, the unknown dynamics of the FOHR system under unknown dynamic environment is locally accurately identified and stored in the form of constant weight neural networks through deterministic learning without dependency of the system parameters. Then, the model-based and learning-based sliding mode controllers based on the identified and stored system dynamics are designed for the similar and new synchronization tasks, respectively. Therefore, the fast synchronization effect is achieved through recalling the empirical dynamics of neurons. Moreover, the control experience can be constantly replenished and stored for reutilization due to the convergence of the identification and synchronization process, which help improves the synchronization speed and accuracy continuously.

## 2. Methods and innovations

**Figure 1:**
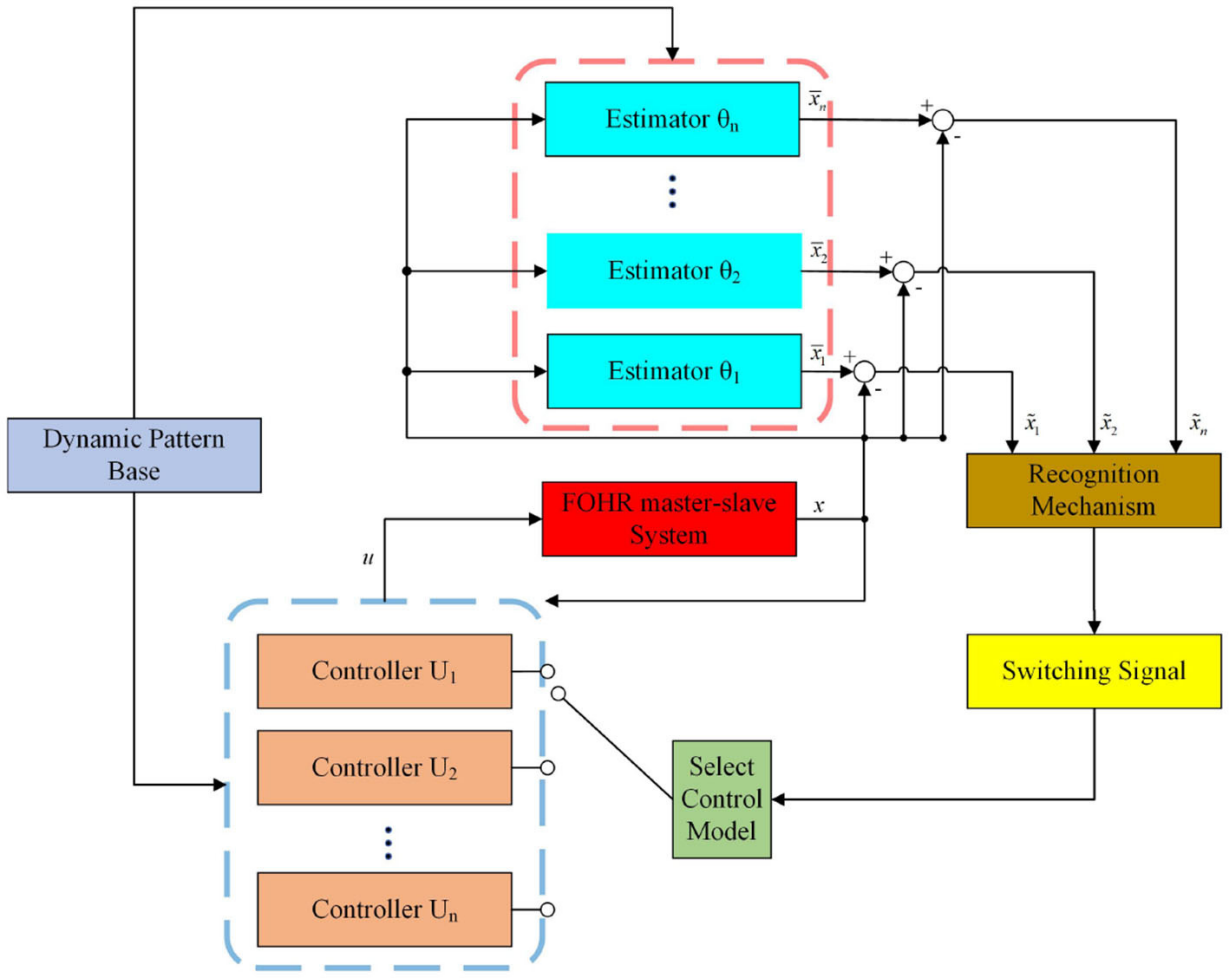
Flowchart of the method.

In this section, the method proposed in this study and the main innovations are briefly introduced. Aiming at the problem of abnormal synchronization of neurons under unknown dynamic environment, the sliding-mode control method is introduced. Different from traditional sliding-mode control policy, the human experience of knowledge acquisition, storage, and re-application is introduced to the control process of our study. Precisely, the unknown dynamic information of the neuron system is identified and stored according to the deterministic learning mechanism by using the RBF neural network. The dynamic information is further applied to the controller to achieve more accurate synchronization effect, which is called the model-based sliding-mode control. Considering the case that the stored dynamic information is limited and the unknown slave system can not be well matched, the relearning-based sliding-mode control is proposed. During this propose, the identified and control experience can be updated and supplemented to the dynamic patter database, which can provide experience for new and unfamiliar synchronization tasks. Thus, the online computing time is shortened, and a better synchronization effect can be achieved. In addition, the problem of excessive chattering faced by traditional sliding mode control method can be solved skillfully by selection small sliding-mode gain by using experiential information. The flowchart of the method is available in Figure 1. The emphasis of our study is not only on the effect of synchronization but also on the efficient storage and reutilization of the experience knowledge in the process of neural system synchronization, which is the essence of intelligent learning and intelligent control and not covered by most existing research studies.

## 3. Preliminary knowledge

### 3.1. Fractional-order definition and basic properties

The fractional order (FO) calculus has a very long mathematical history and has gained extensive attention in the areas of science and engineering with the advent of high computational devices recent years. The FO calculus can be seen as the comprehensive and generalized version of the conventional integer-order calculus, which encompassed both fractional and integer-order differential and integral equations (Dar et al., 2022). Correspondingly, the FO derivative possesses complex or real arbitrary order, for which various mathematical operators have been proposed. Among those operators, the Grunwald-Letnikov (GL) (Huang, 2016), Liouvill (L) (Huang, 2016), Riemamn-Liouville (RL) (Efe, 2009), and Caputo (C) (Gorenflo and Mainardi, 1997) are most commonly used. Compared with the L, RL, and C operators, the GL operator pays more attention to the numerical calculation of fractional-order differentiation. Since the fractional derivative describes memory and hereditary properties in such an appropriate manner that it demonstrates much advantages in system representation compared to the integer-order models, the GL-based fractional order definition (Huang, 2016) is introduced in this study:


(1) $$\begin{array}{l} {}_{a}^{G}D_{t}^{q}f(t)=\frac{d^{q}f(t)}{d{\left(t-a\right)}^{q}}=\underset{h\to 0}{\lim}h^{-q}\sum_{r=0}^{n}{\left(-1\right)}^{r}\;(\begin{array}{c} q\\ r \end{array})f(t-rh), \end{array}$$

where, *G* means the GL based fractional calculus, *nh* = *t*−*a*, if *q* < 0, the Equation (1) is the G-L based fractional integral definition; on the contrary, if *q*>0, the Equation (1) is the G-L based differential definition.

### 3.2. Properties of fractional calculus

The main and commonly used properties of fractional derivatives are given as follows:

1. For *q* = *n*, where *n* is an integer, the operation ${}_{0}D_{t}^{q}f\left(t\right)$ gives the same result as classical differentiation of integer order *n*. Meanwhile, the fractional derivative degenerate to integer derivative.

2. For *q* = 0, the operation becomes the identity operator given as

(2) $$\begin{array}{l} {}_{0}D_{t}^{q}f\left(t\right)=f\left(t\right). \end{array}$$

3. The additive index law (semigroup property)

(3) $$\begin{array}{l} {{}_{0}D_{t}^{\alpha }}_{0}D_{t}^{\beta }f\left(t\right){={}_{0}D_{t}^{\beta }}_{\;0}D_{t}^{\alpha }f\left(t\right){=}_{\;0}D_{t}^{\alpha +\beta }f\left(t\right) \end{array}$$

holds under some reasonable constraints on the function *f*(*t*). In particular, there is


(4) $$\begin{array}{l} D_{t}^{q}\left(D_{t}^{1-q}f\left(t\right)\right)=D_{t}^{1}f\left(t\right)=\frac{d}{dt}f\left(t\right),0<q<1. \end{array}$$

### 3.3. The deterministic learning theory

In 2009, the deterministic learning (DL) theory was proposed for the problem of learning in uncertain dynamic environments (Wang et al., 2009). It mainly focuses on the dynamic process of knowledge learning, representation, and utilization in unknown dynamic environment. With deterministic learning, fundamental knowledge on system dynamics can be accumulated, stored, and represented by constant RBF networks in a deterministic manner. Moreover, in a scenario whereby an adaptive neural network (NN) controller achieves tracking of a periodic or periodic-like reference orbit, the deterministic learning mechanism is shown capable of achieving closed-loop identification of partial system dynamics during tracking control.

In detail, for any unknown continuous non-linear function *f*(*X*): Ω_*X*_→*R* with recurrent system trajectories ψ(*x*_0_), in which $\Omega_{X}\subset R^{q}$ is a compact set, an ideal constant weight vector W^*^ of the RBF networks exists, that is, $f\left(X\right)=W^{*T}\phi \left(X\right)+\varepsilon^{*},\forall X\in \Omega_{X}$, where ε^*^>0 is the approximation error and $X\in \Omega_{X}\subset R^{q}$ denotes the input vector of the radial basic function networks (RBFNs), $W^{*}={\left[w_{1}^{*},\cdots \;,w_{n}^{*}\right]}^{T}\in R^{N}$ is the ideal RBFNs weight with *N* being the number of neurons. $\phi \left(X\right)={\left[\varphi_{1}\left(\left||X-c_{1}|\right|\right),\cdots \;,\varphi_{n}\left(\left||X-c_{n}|\right|\right)\right]}^{T}$ is the regression vector of RBFs with φ_*i*_(·) being one of the radial basic function, and *c*_*i*_ is the center of neurons distributed in the input space. For the radial basic function, the Gaussian function is one of the most commonly used kernel RBFs given as $\varphi_{i}\left(\left||X-c_{i}|\right|\right)=exp\left[\frac{-{\left(X-c_{i}\right)}^{T}\left(X-c_{i}\right)}{\eta_{i}^{2}}\right]$, in which η_*i*_ is the adjacent width of the radial base kernel. It satisfies the Schoenberg theorem (Schoenberg, 1938) and is localized basis function in the sense that φ_*i*_(||*X*−*c*_*i*_||) → 0 as ||*X*|| → ∞. All these properties of the Gaussian function provide a rich source of RBFs that are suitable for interpolation of data in Euclidean spaces. The conditional non-singularity property is essential in proving the partial persistent excitation (PE) condition of RBF networks, which is the key to the accurate identification ability for the deterministic learning theory.

## 4. Dynamic identification of the fractional-order HR model via deterministic learning

### 4.1. The fractional-order HR model

With the development of neuroscience, various differential models have been proposed for describing the neuron system, including the Hodgin-Huxley (HH) model, the FitzHugh-Nagumo (FHN) model, the Hindmarsh-Rose (HR) model, and the Ermentrout neuronal model. Among those models, the HR model, possessing the simplest system form, can accurately describe the signal transmission process across the nerve fiber membrane. Thus, the HR model is commonly used for neuron dynamic describing and analysis. The classical three-variable HR neuronal model can be described by the following equations:


(5) $$\begin{array}{l} \dot{x}=y-ax^{3}+bx^{2}-z+I\;\\ \dot{y}=c-dx^{2}-y,\\ \dot{z}=r\left(s_{0}(x-q_{0})-z\right) \end{array}$$

where *x* is the membrane potential, *y* is the recovery variable standing for the gating dynamics of the potassium (*K*^+^) channel, and *z* represents the adaptation current corresponding to the dynamics of calcium (*Ca*^2+^) channel. Moreover, the model parameters *a, b, c, r*, and *s*_0_ are positive constants, while the parameter *q*_0_ stands for the resting potential, and *I* represents the external stimulation input.

The FO differential model has more advantages in neuronal dynamic description compared to that of the integer-order model. In addition, the FO system has a wider stability region. Thus, in this study, the following fractional order HR (FOHR) neuronal model is introduced, that is,


(6) $$\begin{array}{l} D_{t}^{q}x=y-ax^{3}+bx^{2}-z+I\\ D_{t}^{q}y=c-dx^{2}-y,\\ D_{t}^{q}z=r(s_{0}(x-q_{0})-z) \end{array}$$

in which, the operator $D_{t}^{q}$ represents the GL fractional derivative as shown in Equation (1).

The state variables and model parameters of the FOHR model possess the same physical meaning with the integer-order HR model. Through bifurcation analysis under different values of the external stimulation input I and fractional order q, the FOHR model demonstrates a wealth of dynamic behaviors, such as the subthreshold oscillations, spiking, bursting as well as chaotic behaviors.

**Figure 2:**
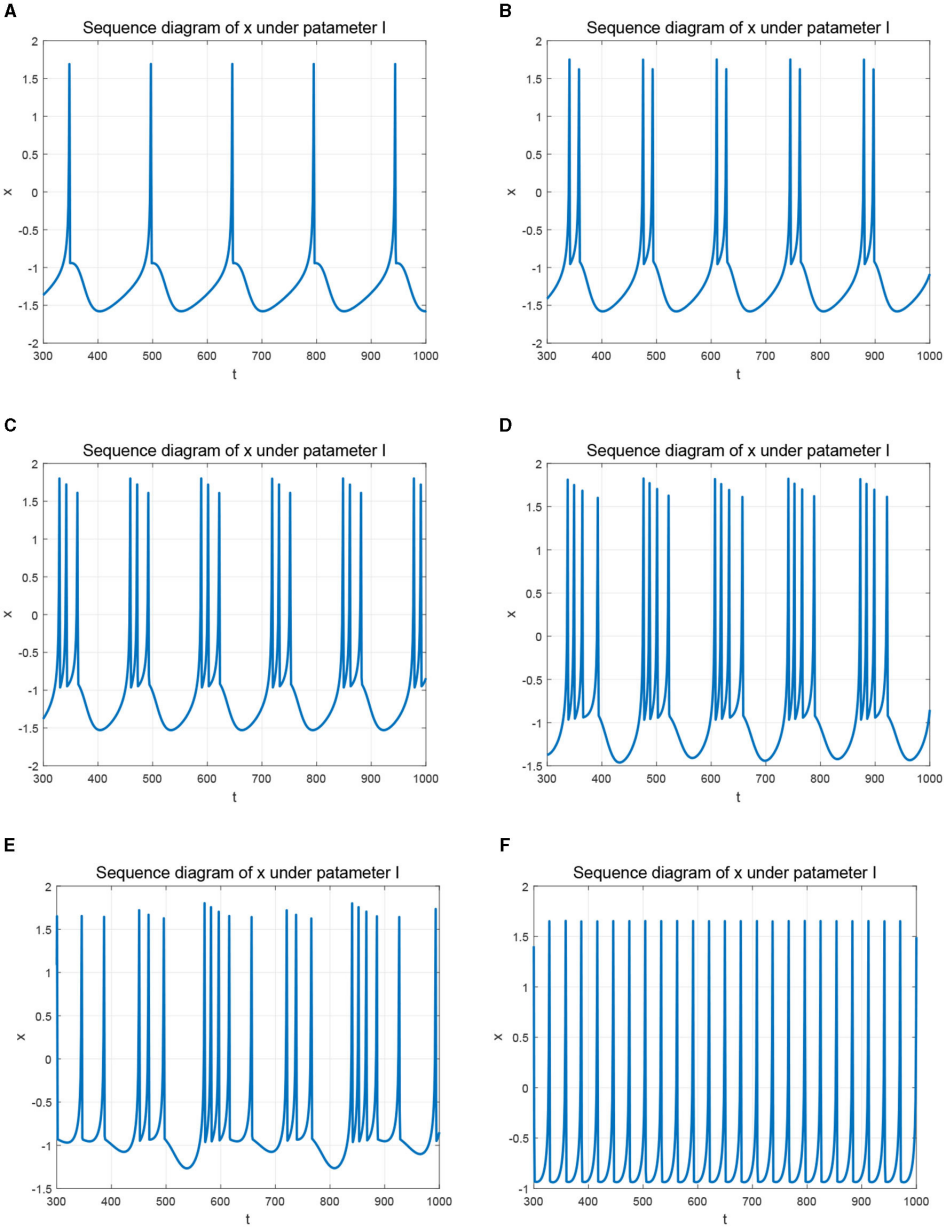
Time response of membrane potential *x* with different parameters *I*. **(A)** *I* = 1.5. **(B)** *I* = 1.8. **(C)** *I* = 2.3. **(D)** *I* = 2.8. **(E)** *I* = 3.2. **(F)** *I* = 3.58.

In detail, when taking the fractional order *q* = 1, the FOHR model degenerates to an integer-order HR model. By setting *q* = 1 and the corresponding system parameters *a* = 1.0, *b* = 3.0, *c* = 1.0, *d* = 6.0, *r* = 0.013, *s* = 4.0, and *q*_0_ = −1.56, diverse non-linear dynamics under different external stimulus I of the HR model are generated. By changing the control parameter *I*, the membrane potential *x* presents different state characteristics, which can be seen from Figure 2, in which the initial system state (*x*_0_, *y*_0_, *z*_0_) is set as (0.1, 1.0, 0.2).

Precisely, when setting *I* = 1.5, the neuron produces slow-peak regular spiking (single-cycle spiking) state as given in Figure 2A. Gradually increasing *I* to 1.8, 2.3, 2.8, the HR system exhibits regular bursting state, shown as the period-2, period-3, and period-4 bursting behaviors, respectively, which can be seen from Figures 2B–D. When *I* increased to 3.2, the state *x* of the HR system becomes chaotic as shown in Figure 2E. Further increasing *I* to 3.58, the system regresses to a fast single-cycle spiking state as demonstrated in Figure 2F, in which the period interval is significantly shorter and the rate of the dynamic activity is much faster than that of the interval demonstrate in Figure 2A.

### 4.2. The dynamic behavior of the FOHR model under fractional order *q*

Except for the non-linear behavior of the time response of the membrane potential *x*, the inter-spike interval (*ISI*) (Rabinovich and Abarbanel, 1998) is one of most commonly used physiological indicators, which carries important information of neuronal firing. In the following discussion, the bifurcation diagram of the peak membrane potential *x*_*max*_ and the *ISI* sequence of the FOHR neural system with different bifurcation parameters are considered.

**Figure 3:**
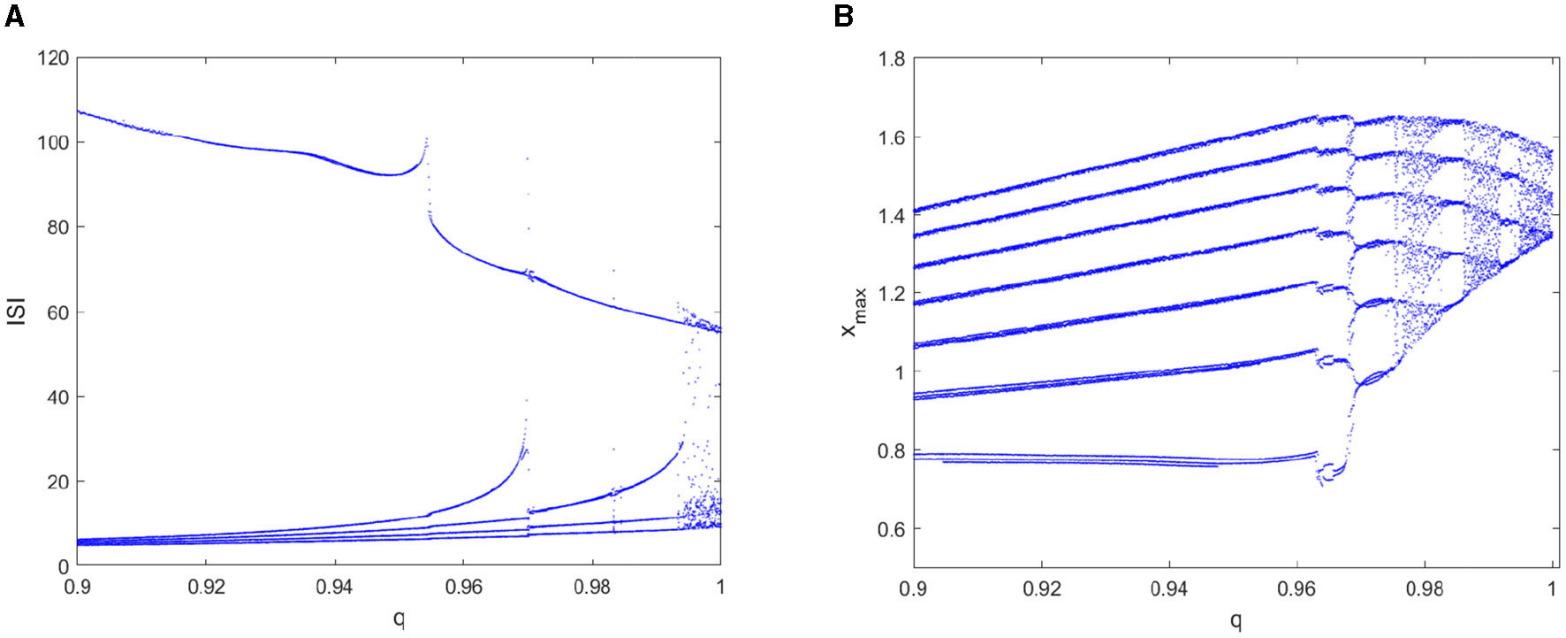
Bifurcation diagram of the *ISI* and the *x*_*max*_ sequence with parameter *q*. **(A)** bifurcation diagram of *ISI*. **(B)** bifurcation diagram of *x*_*max*_.

First, the dynamic non-linearity of the FOHR model under different fractional orders *q* with the external excitation *I* = 3 is considered. As shown in Figure 3A, the bifurcation diagram of the *ISI* sequence exhibits a comb-shaped region with the increase of fractional order *q*. Correspondingly, the bifurcation diagram of the peak of the membrane potential *x* (denoted as *x*_*max*_) demonstrates that the discharge characteristics of the system varies more obviously according to the fractional order. That is, with the increase of the fractional order *q* within a certain range, the system as a whole shows the tendency of periodic decline, and the periodic bursting phenomenon occurs as demonstrated in Figure 3B. In other words, the firing behavior of neurons becomes more complex and unstable with the increase of the fractional order of the neuronal system, exhibiting richer dynamic activity characteristics.

### 4.3. The dynamic behavior of the FOHR model under external excitation *I*

Second, take the external excitation *I* as the control parameter for analyzing the dynamic behaviors of the FOHR model with a certain fractional order. The parameter *I* is taken within the interval [1.2, 4.3] and *r* = 0.013.

**Figure 4:**
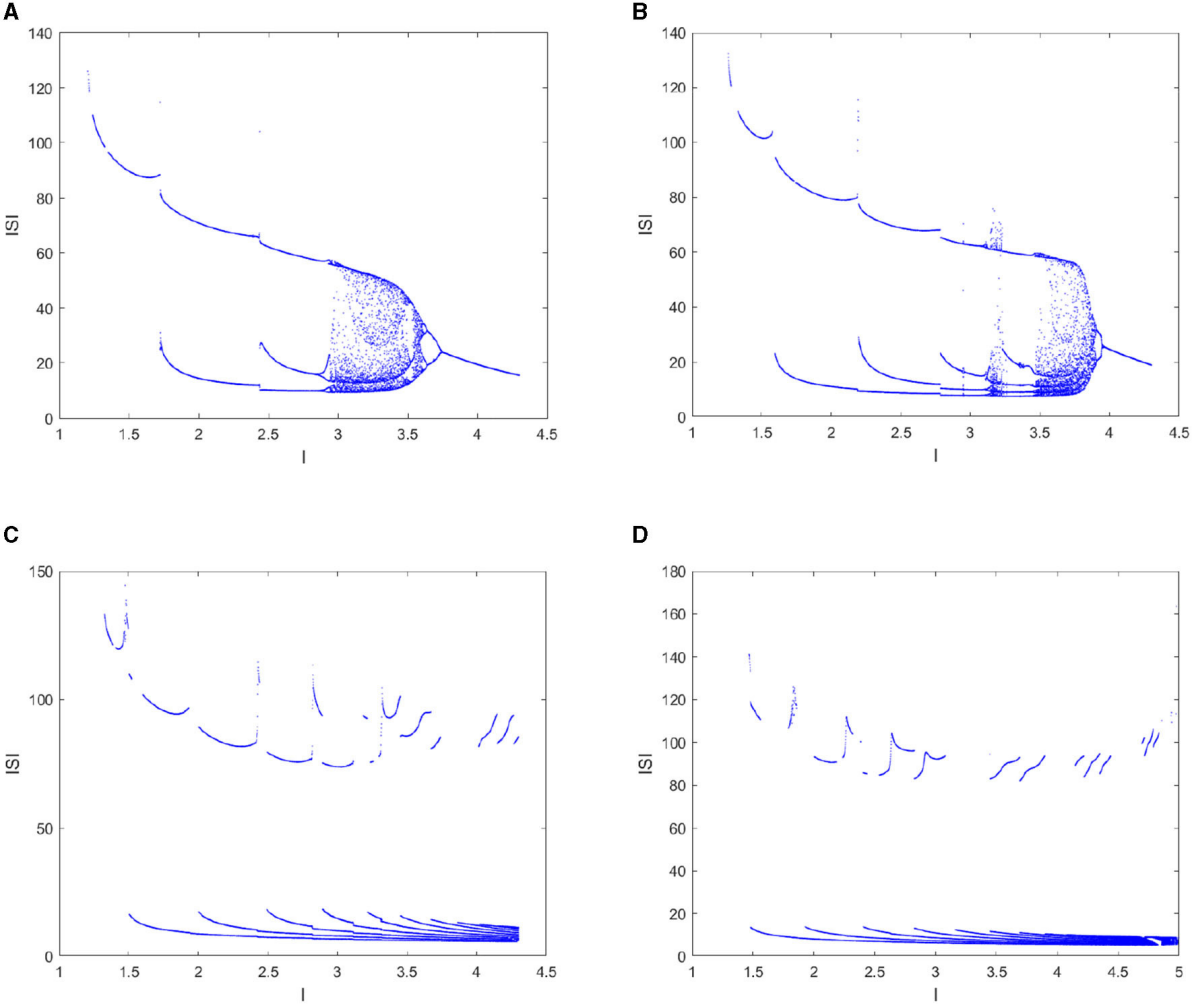
Bifurcation diagram of the *ISI* sequence with parameter *I*. **(A)** *q* = 1. **(B)** *q* = 0.98. **(C)** *q* = 0.96. **(D)** *q* = 0.95.

The simulation results of the bifurcation diagram of the *ISI* sequence (shown in Figure 4) exhibit that the dynamic characteristics of the FOHR system become more complex with the increase of I. Taking the integer order as an example (given in Figure 4A), the *ISI* sequence experiences the process of period-2 bifurcation to chaos and then back to single period by the period-doubling bifurcation process. Correspondingly, the *ISI* sequence of the 0.98-order FOHR model indicates similar discharge behaviors with that of the integer-order model, but the chaos duration is reduced. In addition, from the amplitude of the *ISI* sequence, it can be seen that the effect of external stimulus current on the system dynamics was much obvious.

**Figure 5:**
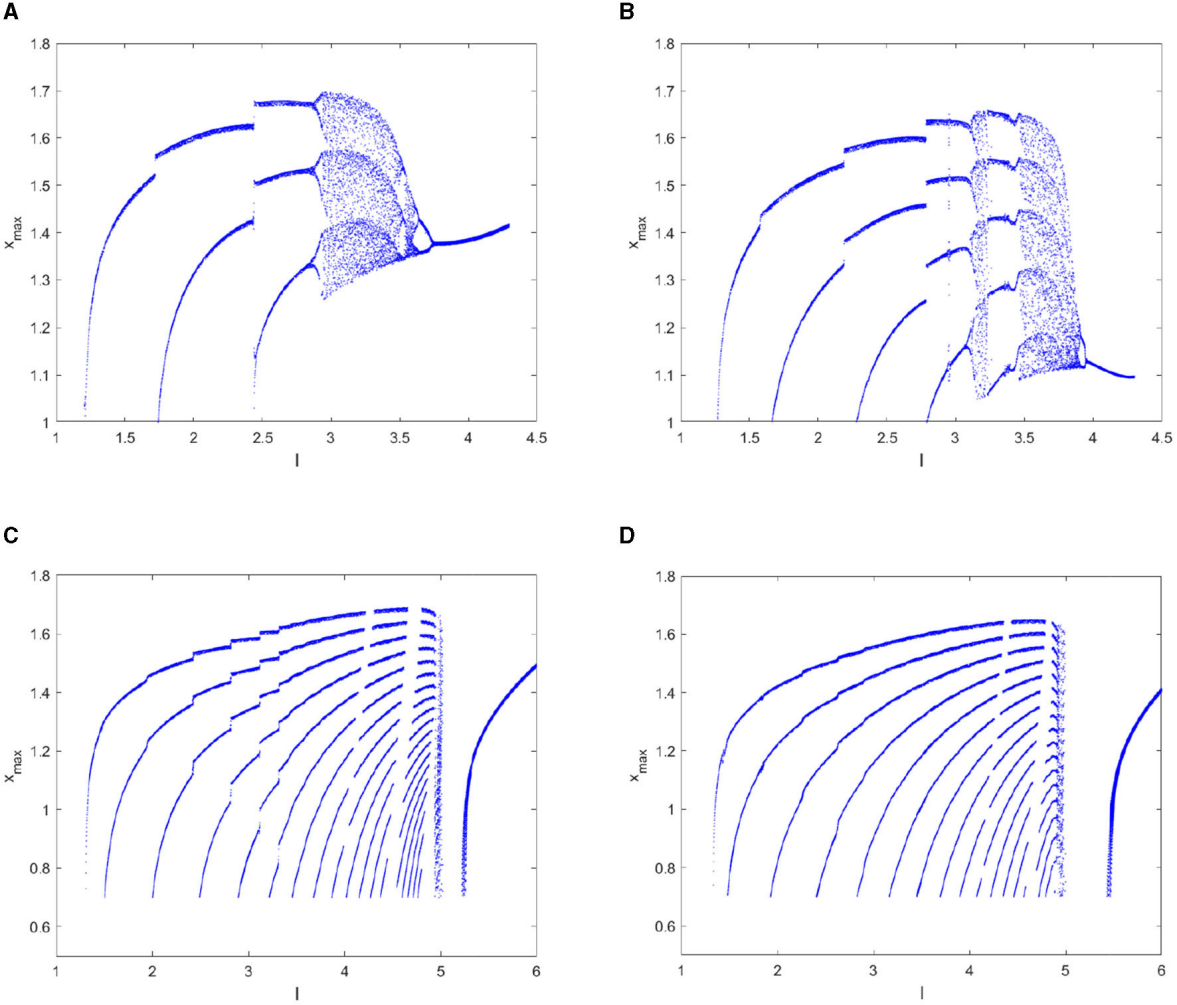
Bifurcation diagram of the *x*_*max*_ sequence with parameter *I*. **(A)** *q* = 1. **(B)** *q* = 0.98. **(C)** *q* = 0.96. **(D)** *q* = 0.95.

According to the bifurcation diagram of the *x*_*max*_ sequence shown in Figure 5, the dynamical behaviors correspond to the same fractional order has similar and abundant dynamic characteristics with that of the *ISI* sequence. In addition, some hidden information contained in the integer order can be clearly demonstrated in the 0.98-order HR model as shown in Figure 5B, such as the period-4 cluster bursting under *I* = 3.0, the period-5 cluster discharge when *I* = 3.3, the comb-shaped region and the chaotic region. If a further decrease in the fractional order *q* to 0.96 and 0.95 as can be seen from Figures 5C, D, the dynamic structure of the FOHR system changes qualitatively. Precisely, with the increase of parameter *I*, the dynamic behavior of the FOHR model becomes more complex. The structure and stability of the system is influenced correspondingly.

### 4.4. The dynamic behavior of the FOHR model under parameter *r*

**Figure 6:**
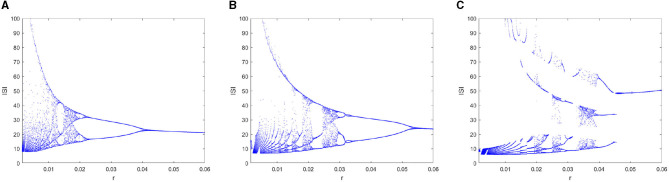
Bifurcation diagram of the *ISI* sequence with parameter *r*. **(A)** *q* = 1. **(B)** *q* = 0.98. **(C)** *q* = 0.96.

**Figure 7:**
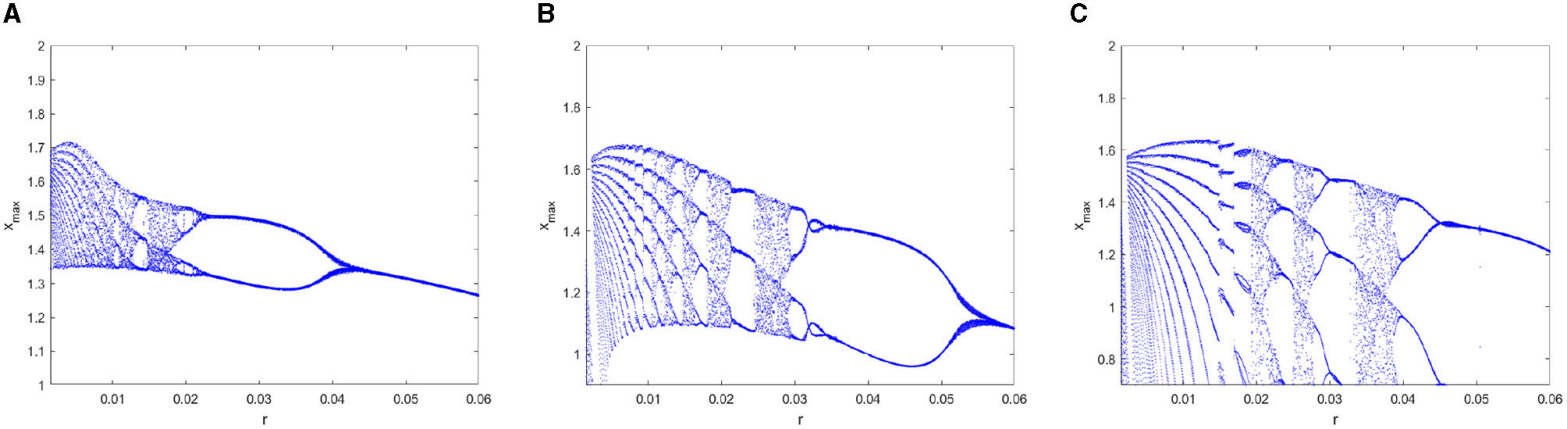
Bifurcation diagram of the *x*_*max*_ sequence with parameter *r*. **(A)** *q* = 1. **(B)** *q* = 0.98. **(C)** *q* = 0.96.

To further analyze the dynamic characteristics of the FOHR model, another important parameter *r* which relates to the calcium (*Ca*^2+^) concentration and significant to many neurological disorders, is considered as the control parameter in this part. All the other parameters are kept as the same as mentioned above, while parameter *I* is fixed to 3.5. When ranging the parameter *r* from 0.0015 to 0.06, a variety of dynamic behaviors of the FOHR neuron system are presented. As shown in Figures 6, 7, the bifurcation diagram of the *ISI* sequence and the *x*_*max*_ sequence demonstrate similar non-linear characteristics. Moreover, compared to the integer-order HR model, the 0.98-order HR model presents a more detailed and clear dynamic process.

In conclusion, the dynamic simulations given above suggest that compared to the integer-order HR model, the fractional-order HR model can describe the numerous computational features and the non-linear dynamics of the neuron model more accurately, which help enrich the functional neuron mechanisms and further ensures more accurate dynamic analysis. Thus, it is necessary and important to introduce the FOHR model, and the FOHR model with fractional order *q* = 0.98 is taken into consideration in the following study.

## 5. Identification of the FOHR model via deterministic learning

The above numerical simulations are obtained based on the assumption that the parameters of neurons are known, which is also commonly used in most related research studies. However, it is too ideal for most practical neuron systems. More precisely, the neuron parameters are actually unknown and vary dynamically with the dynamic environment. Therefore, how to identify the non-linear dynamics of the neuronal model under unknown dynamic environment is essential for comprehensive understanding of the non-linear characteristics of the actual HR model. This will be the focus of the discussion below.

To identify the unknown system dynamics of the fractional order HR model, the RBF neural network is considered:


(7) $$\begin{array}{l} f_{i}\left(x;\mu \right)={Ŵ}_{\xi_{i}}^{T}\phi_{\xi_{i}}\left(x\right), \end{array}$$

where $x\in \Omega_{f}\subset R^{n}$ is the neural networks (NNs) input, ${Ŵ}_{\xi }={\left[{Ŵ}_{\xi_{1}},\cdots \;,{Ŵ}_{\xi_{n}}\right]}^{T}$ is the estimate of the ideal weight matrix, and $\phi_{\xi }\left(x\right)={\left[\varphi_{\xi_{1}}\left(x\right),\cdots \;,\varphi_{\xi_{n}}\left(x\right)\right]}^{T}$ is chosen as a vector of Gaussian functions, that is given as


(8) $$\begin{array}{l} \varphi_{\xi_{i}}\left(x\right)=exp\left(\frac{-||X-c_{i}||}{\eta_{i}^{2}}\right),i=1,\cdots \;,N_{i}, \end{array}$$

where *c*_*i*_ denotes the center vector of the *ith* basis function and η_*i*_ is the adjacent width of the basis function, (·)_ξ_ represents the neurons that was distributed close to the system trajectory, which plays the main role during the process of the neuronal dynamic identification.

Since the input of the RBFNN possesses regression property, the RBF NNs can locally accurately approximate the non-linear function along the system trajectory, that is,


(9) $$\begin{array}{l} f_{i}\left(x;\mu \right)=W_{\xi_{i}}^{*T}\varphi_{\xi_{i}}\left(x\right)+\varepsilon_{i_{1}}, \end{array}$$

where $W_{\xi_{i}}^{*}$ is the optimal weights vector and ε_*i*_1__ is the bounded identification error close to zero.

The dynamic investigations and simulations of the HR model discussed above have revealed the regression characteristics of the neuron system. It is the regression property of the HR model that reminds us of the deterministic learning theory, which emphasizes that almost any period or period-like (recurrent) NN input can lead to the satisfaction of the partial persistent excitation condition (PE) along the system trajectory by using the localized RBFNs. Furthermore, the identified system dynamics can be stored due to the convergence of the NN weights; that is,


(10) $$\begin{array}{l} {\bar{W}}_{i}=mean_{t\in \left[t_{a},t_{b}\right]}{Ŵ}_{i}\left(t\right), \end{array}$$

where *t*_*a*_>*t*_*b*_>0 is the time segment referring to a piece of time segment within the convergence process of the NN weights and "mean" is the arithmetic mean. Then, the unknown system dynamics can be accurately identified and stored by the constant vector of neural networks, giving as


(11) $$\begin{array}{l} f_{i}\left(x;\mu \right)={\bar{W}}_{\xi_{i}}^{T}\varphi_{\xi_{i}}\left(x\right)+\varepsilon_{i_{2}}, \end{array}$$

where $\varepsilon_{i_{2}}=\varepsilon_{i_{1}}-{\tilde{W}}_{\xi_{i}}^{T}\varphi_{\xi_{i}}\left(x\right)$ is the practical approximation error of the system dynamics by using the constant NN vector ${\bar{W}}_{\xi_{i}}^{T}\varphi_{\xi_{i}}$ with ${\tilde{W}}_{i}={Ŵ}_{i}-W_{i}^{*}$ being a small positive number approaching zero.

For different dynamic external excitation *I* of the FOHR system under the given fractional order, different state trajectories are generated. Based on the approximate process by using the DL method, accurate identification of unknown system dynamics *f*_*i*_(*x*; μ) are obtained and stored as constant RBF neural networks ${\bar{W}}_{\xi i}^{T}\varphi_{\xi i}^{T}$. Then, a certain number of constant RBF neural networks compose a pattern base which denoted as $\chi =\left\{\chi^{k}={\bar{W}}_{i}^{kT}|k=1,\cdots \;,K\right\}$.

***Remark 1:*** The process of system dynamics identification and storage of the FOHR system in unknown dynamic environment will simulate the way of human learning and memorizing new knowledge. The created pattern base, that is, the memory in the mind of knowledge, can be directly invoked in the control process.

## 6. Sliding mode control of the FOHR system by using deterministic learning

### 6.1. Problem description

In this sub-section, the model-based sliding mode control problem of two FOHR neuronal models is considered. The two neurons interconnect in a master-slave configuration. The master FOHR neuronal model is given as follows:


(12) $$\begin{array}{l} D_{t}^{q}x_{m,1}=x_{m,2}-ax_{m,1}^{3}+bx_{m,1}^{2}-x_{m,3}+I,\;\\ D_{t}^{q}x_{m,2}=c_{m}-dx_{m,1}^{2}-x_{m,2},\;\\ D_{t}^{q}x_{m,3}=r(s_{0}(x_{m,1}-q_{0})-x_{m,3}), \end{array}$$

and the slave FOHR neuronal model under control is denoted by


(13) $$\begin{array}{l} D_{t}^{q}x_{s,1}=x_{s,2}-ax_{s,1}^{3}+bx_{s,1}^{2}-x_{s,3}+I+d_{1}+u_{1},\\ D_{t}^{q}x_{s,2}=c_{s}-dx_{s,1}^{2}-x_{s,2}+d_{2}+u_{2},\\ D_{t}^{q}x_{s,3}=r(s_{0}(x_{s,1}-q_{0})-x_{s,3})+d_{3}+u_{3}, \end{array}$$

where *d*_*i*_, *i* = 1, 2, 3 represents the bounded unknown external disturbance; that is, $|d_{i}|\leq \bar{d_{i}},i=1,2,3$ and the terms *u*_*i*_ and *i* = 1, 2, 3 denote the control inputs of the state variables.

For the convenience of discussion, the simplified master-slave neuron system models are presented as follows:


(14) $$\begin{array}{l} \begin{array}{c} {\;D}_{t}^{q}x_{m}=f_{m}\left(x_{m}\right),\\ D_{t}^{q}x_{s}=f_{s}\left(x_{s}\right)+d_{i}+u_{i}, \end{array} \end{array}$$

where $x_{m}={\left[x_{m,1},x_{m,2},x_{m,3}\right]}^{T}$ and $x_{s}={\left[x_{s,1},x_{s,2},x_{s,3}\right]}^{T}$ are the state vectors of the master and slave neuronal system, respectively. *f*_*m*_ represents the known system dynamics vectors of the master FOHR model. Correspondingly, *f*_*s*_ represents the unknown system dynamics vectors of the slave FOHR model. Precisely, *f*_*s*_ is smooth, but unknown non-linear dynamics of the slave system. *d*_*i*_ and *u*_*i*_ have the same meaning as the formula given in Equation (13). The main task in this part is to realize the synchronization of the master-slave system with proper amount of calculation and correct the synchronization error by adjusting the parameters.

### 6.2. Model-based sliding mode control of the FOHR system

The synchronization of the master-slave neuronal system is to drive the slave neuron system to track the state as well as the trajectory of the master system under certain external disturbance in unknown dynamic environment by properly designed controller. In order to achieve ideal stability effect of the control system, the gain parameters of the traditional sliding mode control algorithm are usually set too large, which leads to serious chattering problem. In this part, the obtained system dynamics ${\bar{W}}_{i}$ stored in the pattern base χ is applied for the sliding mode control to achieve fast synchronization performance for the master-slave neuron system. In addition, the accurate modeling of the system dynamics help reduce the synchronization error of the master-slave system without large gain, thus reducing the chattering caused by sliding mode gain.

The synchronization error of the master-slave FOHR system is defined as follows;


(15) $$\begin{array}{l} \begin{array}{l} \begin{array}{l} \;e_{i}=x_{s,i}-x_{m,i},\;\\ D_{t}^{q}e_{i}=f_{s,i}(x)+d_{i}+\mu_{i}-f_{m,i}(x), \end{array} \end{array} \end{array}$$

where *i* = 1, 2, 3. To achieve fast synchronization of the master-slave FOHR system, the identified and stored model-based sliding mode control method is proposed. First, the fractional order proportional integral sliding surface is designed as follows:


(16) $$\begin{array}{l} \;s_{i}=c_{i}D_{t}^{1-q}e_{i}+e_{i},\\ D_{t}^{q}s_{i}=c_{i}e_{i}+D_{t}^{q}e_{i}. \end{array}$$

where *s*_*i*_, (*i* = 1, 2, 3) is the fractional order proportional integral sliding surface. The derivative of the sliding mode surface can be achieved according to the properties of fractional order models discussed in Section 3.2, that is,


(17) $$\begin{array}{l} {ṡ}_{i}=c_{i}e_{i}+f_{s,i}\left(x\right)+d_{i}+u_{i}-f_{m,i}\left(x\right), \end{array}$$

where the corresponding constant rate of convergence is designed as


(18) $$\begin{array}{l} {ṡ}_{i}=-\eta_{i}sgn\left(s_{i}\right). \end{array}$$

The following sliding mode control rate is designed according to the Equations (17) and (18)


(19) $$\begin{array}{l} \mu_{i}=-\eta_{i}sgn\left(s_{i}\right)-c_{i}e_{i}+f_{m,i}\left(x\right)-f_{s,i}\left(x\right). \end{array}$$

For the unknown system dynamics *f*_*m,i*_(*x*) of the slave system, the rapid recognition process is introduced, that is,


(20) $$\begin{array}{l} {\dot{\bar{x}}}_{i}^{k}=-b_{i}\left({\bar{x}}_{i}^{k}-x_{i}\right)+{\bar{W}}_{i}^{kT}\varphi_{i}\left(x\right),k=1,\cdots \;,K, \end{array}$$

in which ${\bar{x}}_{i}^{k}$ represents the state of the dynamic model and the corresponding dynamic information of the system has been identified and stored in the pattern base χ as mentioned above, *x*_*i*_ is the *ith* state of the unknown slave system, and *b*_*i*_>0 is a design parameter.

For the unknown slave FOHR system, the recognition error system is given as


(21) $$\begin{array}{l} {\dot{\tilde{x}}}_{i}^{k}=-b_{i}{\tilde{x}}_{i}^{k}+\left({\bar{W}}_{i}^{kT}\varphi_{i}\left(x\right)-f_{s,i}\left(x\right)\right),i=1,\cdots \;,n, \end{array}$$

where ${\tilde{x}}_{i}^{k}={\bar{x}}_{i}^{k}-x_{i}$ is the state tracking error between the empirical pattern stored in the base and the unknown slave system.

Commonly, without identifying the unknown dynamics of the unknown slave FOHR system, the differences between the dynamic systems stored in the pattern base and the slave pattern denoted as $\left|{\bar{W}}_{i}^{kT}\varphi_{i}\left(x\right)-f_{s,i}\left(x\right)\right|$ shown in Equation (21) is unavailable for direct computation. However, as presented in Wang et al. (2009), the state tracking error $\left|{\tilde{x}}_{i}^{k}\right|$ can be explicitly measured.

For any unknown slave FOHR system with regression system trajectory φ(*x*_*d*0_), the tracking error $\left|{\tilde{x}}_{i}^{k}\right|$ can be achieved within finite time by properly selecting the design parameters; that is, by introducing the average *L*_1_-norm based dynamic similarity measure, that is given as


(22) $$\begin{array}{l} \begin{array}{l} ||{\tilde{x}}_{i}^{k}(t)|{|}_{1}=\frac{1}{T}\int_{t}^{t+T}|{\tilde{x}}_{i}^{k}(\tau )|d\tau , \end{array} \end{array}$$

where *T*>0 is a design parameter, and the difference between system dynamics can be explicitly measured. Based on the similarity measure, the smallest tracking error between certain unknown slave system and the system identified as well as stored in the pattern base χ can be obtained, that is


(23) $$\begin{array}{l} x_{i}^{0}=\min(\|{\tilde{x}}_{i}^{k}\|,k=1,\cdots ,m), \end{array}$$

in which *m* denotes the number of models stored in the pattern base χ.

***Remark 2***: According to the recognition process discussed above, the dynamic differences between the slave system and those systems stored in the pattern base can be accurately measured without identifying of the dynamic information of the slave system. This process is therefore referred to as rapid recognition. In particular, the most similar dynamic pattern $\chi^{k_{0}}$ can be selected from the pattern base according to the minimum recognition error, and the dynamic information of the selected model denoted as ${\bar{W}}_{i}^{k_{0}T}$ can be used to replace the unknown dynamics *f*_*s,i*_(*x*) of the slave FOHR model in the following control process.

Based on the recognition process, the unknown system dynamics *f*_*s,i*_(*x*) of the slave system can be locally accurately identified as well as stored by the constant weight NNs along the system trajectory, that is,


(24) $$\begin{array}{l} f_{s,i}\left(x\right)={\bar{W}}_{i}^{k_{0}T}\varphi_{i}\left(x\right)+\varepsilon_{i_{2}}. \end{array}$$

Substituting Equation (24) into Equation (19), the following control rate is obtained:


(25) $$\begin{array}{l} u_{i}=-\eta_{i}sgn\left(s_{i}\right)-c_{i}e_{i}+f_{m,i}\left(x\right)-{\bar{W}}_{i}^{k_{0}T}\varphi_{i}\left(x\right), \end{array}$$

where ${\bar{W}}_{i}^{k_{0}T}\varphi_{i}\left(x\right)$ denotes the most similar dynamic model recognized from the pattern base to the unknown slave system by using the localized RBFNNs located close to the system trajectory.

***Remark 3***: The mode-based sliding mode control is designed to fit the unknown dynamics of the slave system quickly by calling the acquired dynamic information of the neurons, and the experience is applied to the control process. During this process, the generalization ability of the rapid recognition mechanism based on deterministic learning provides the right decisions for invoking right dynamic patterns for better control performance. Put it another way, the empirical dynamic information learned and stored in the pattern base is so sufficiently utilized that the on-line control time is reduced and the fast synchronization is achieved. Compared with the traditional sliding mode control method, the model based sliding mode control algorithm can effectively reduce the sliding mode gain so as to reduce the chattering problem of the system.

### 6.3. Stability analysis

To verify the stability of the master-slave synchronization control system, consider the following Lyapunov function candidate:


(26) $$\begin{array}{l} V_{i}=\frac{1}{2}s_{i}^{2}. \end{array}$$

The derivative of *V* is


(27) $$\begin{array}{l} {\dot{V}}_{i}=s_{i}{ṡ}_{i}. \end{array}$$

By taking the differential equation of the sliding surface given in Equation (17) and the sliding mode rate given in Equation (25) to Equation (27), we have


(28) $$\begin{array}{l} {\dot{V}}_{i}=s_{i}(c_{i}e_{i}+f_{s,i}(x)+d_{i}+u_{i}-f_{m,i}(x)),\;\\ \;=s_{i}(f_{s,i}(x)+d_{i}-\eta_{i}sgn(s_{i})-{\bar{W}}_{i}^{k_{0}T}\varphi_{i}(x)).\\ \;=s_{i}(\varepsilon_{i2}+d_{i}-\eta_{i}sgn(s_{i})), \end{array}$$

as shown in Equation (28), the external disturbance *d*_*i*_ and the identification error ε_*i*2_ have an upper bound. Therefore, to ensure that the function *V*_*i*_ is negative definite, just need to set appropriate sliding mode gain η_*i*_ to make the equation η_*i*_ > ε_*i*2_ + *d*_*i*_ work, which will further ensure the convergence of synchronization error.

### 6.4. Relearning-based sliding mode control of the master-slave FOHR system

As discussed above, the robustness and generalization ability of the recognition system are greatly related to the richness of the patterns in the dynamic pattern database. When considering the condition that there is no ideal similar dynamic pattern in the pattern base for the unknown slave system, that is,even if the smallest tracking error exists, the corresponding constant system dynamics ${\bar{W}}_{i}^{k_{0}T}\varphi_{i}\left(x\right)$ utilized in the control rate may result in large synchronization error and affects the stability and convergence of the control process. This analysis suggests that it is necessary to further explore how to improve the synchronization effect under limited off-line pattern base.

In order to solve the above problems to ensure a stable and rapid control effect, further identification of the unknown slave system is considered. Based on the selected dynamics ${\bar{W}}_{i}^{k_{0}T}\varphi_{i}\left(x\right)$ according to the smallest recognition error, the improved control rate is proposed below:


(29) $$\begin{array}{l} \;u_{i}=-\eta_{i}sgn(s_{i})-c_{i}e_{i}+f_{m,i}(x)-{\hat{W}}_{i}^{T}\varphi_{i}(x),\\ u_{i}(0)\;=-\eta_{i}sgn(s_{i})-c_{i}e_{i}+f_{m,i}(x)-{\bar{W}}_{i}^{k_{0}T}\varphi_{i}(x). \end{array}$$

Based on the Equation (29) and Equation (17), the time derivative of the sliding mode variable is given as


(30) $$\begin{array}{l} {ṡ}_{i}=-{\tilde{W}}_{i}^{T}\varphi_{i}\left(x\right)+\varepsilon_{i}-\eta_{i}sgn\left(s_{i}\right)+d_{i}, \end{array}$$

where $-{\tilde{W}}_{i}^{T}\varphi_{i}\left(x\right)=f_{s,i}\left(x\right)-{Ŵ}_{i}^{T}\varphi_{i}\left(x\right)$, |*d*_*i*_(*t*)| ≤ *D*_*i*_, and $|\varepsilon_{i}|\leq \bar{\varepsilon }$ are external excitation and identification error with upper bound, respectively. For convenience of presentation, define $D_{i}+{\bar{\varepsilon }}_{i}=\kappa_{i}$, the derivation of the sliding mode surface is given as follows:


(31) $$\begin{array}{l} \dot{s_{i}}=-{\tilde{W_{i}}}^{T}\varphi_{i}\left(x\right)-\eta_{i}sgn\left(s_{i}\right)+\kappa_{i}. \end{array}$$

In addition, the NN adaptive update law of the sliding mode control is designed as


(32) $$\begin{array}{l} \dot{\hat{W_{i}}}=\dot{\tilde{W_{i}}}=\Gamma_{i}\varphi_{i}\left(x\right)s_{i}-\sigma_{i}\Gamma_{i}|s_{i}|{Ŵ}_{i}, \end{array}$$

where Γ_*i*_ and σ_*i*_ are positive adjustable parameters. Since $\kappa_{i}=D_{i}+\bar{\varepsilon }$, the synchronization error is precisely related to the identification accuracy; that is, the higher the identification accuracy of the unknown slave system, the better the synchronization effect of the master-slave neuronal system.

***Theorem 1*** Consider the master-slaver neuron FOHR system as shown in Equation (14), the learning-based controller Equation (29), and the NN weight updating law Equation (32). For initial condition *x*_*d*_(0) which generates the recurrent orbit φ_*d*_(*x*_0_), and with corresponding initial condition *x*(0) selected in a close vicinity of the recurrent orbit, the control error of the master-slave system described by Equation (15) converges exponentially to a small neighborhood around zero.

***Proof***: For the sliding mode-based control system, consider the following Lyapunov function:


(33) $$\begin{array}{l} V=\frac{1}{2}s_{i}^{2}+\frac{1}{2}{\tilde{W}}_{i}^{T}\Gamma^{-1}{\tilde{W}}_{i}. \end{array}$$

The derivative of V is


(34) $$\begin{array}{l} {\dot{V}}_{i}=s_{i}{ṡ}_{i}+{\tilde{W}}_{i}^{T}\Gamma_{i}^{-1}\dot{\tilde{W_{i}}}. \end{array}$$

By introducing the designed sliding mode surface and the adaptive update rate equation, there is,


(35) $$\begin{array}{l} {\dot{V}}_{i}=s_{i}(-{\tilde{W}}_{i}^{T}\varphi_{i}(x)-\eta_{i}sgn(s_{i})+\kappa_{i})+{\tilde{W}}_{i}^{T}\Gamma_{i}^{-1}(\Gamma_{i}\varphi_{i}(x)s_{i}\\ \;-\sigma_{i}\Gamma_{i}|s_{i}|{\hat{W}}_{i}),\\ \;=s_{i}\kappa_{i}-s_{i}\eta_{i}sgn(s_{i})-\sigma_{i}{\tilde{W}}_{i}^{T}|s_{i}|{\hat{W}}_{i},\\ \;\leq |s_{i}|(\kappa_{i}-\eta_{i}-\sigma_{i}{\tilde{W}}_{i}^{T}{\hat{W}}_{i}), \end{array}$$

in which,


(36) $$\begin{array}{l} \sigma_{i}{\tilde{W}}_{i}^{T}{\hat{W}}_{i}\leq -\sigma_{i}\|\tilde{W_{i}}{\|}^{2}+\sigma_{i}\|\tilde{W_{i}}\|\;\|W_{i}^{*}\|,\;\\ \;\leq -\frac{\sigma_{i}}{2}\|{\tilde{W}}_{i}{\|}^{2}+\frac{\sigma_{i}}{2}\|{\bar{\bar{W}}}_{i}{\|}^{2}, \end{array}$$

with ${\bar{\bar{W}}}_{i}$ being the upper bound of the ideal identification NN weight $W_{i}^{*}$. Thus, it follows that


(37) $$\begin{array}{l} {\dot{V}}_{i}\leq |s_{i}|\left(\kappa_{i}-\eta_{i}-\frac{\sigma_{i}}{2}||{\tilde{W}}_{i}|{|}^{2}+\frac{\sigma_{i}}{2}||\bar{\bar{W_{i}}}|{|}^{2}\right). \end{array}$$

It is clear that $\dot{V}$ is negative definite when the following conditions are met:


(38) $$\begin{array}{l} |\eta_{i}|>\frac{\sigma_{i}}{2}||\bar{\bar{W_{i}}}|{|}^{2}+\kappa_{i}or\;||{\tilde{W}}_{i}||>\frac{\sigma_{i}}{2}||{\bar{\bar{W}}}_{i}||+\sqrt{\frac{2\kappa_{i}}{\sigma_{i}}}. \end{array}$$

Since the ideal identification NN weight $W_{i}^{*}$, the external excitation *d*_*i*_ and the estimate error ε_*i*_ are all upper bounded; therefore, all signals in a closed-loop control system remain bounded, including the estimate NN weight Ŵ_*i*_ and the sliding mode variable *s*_*i*_.

In addition, to the convergence of the sliding mode variable, the following Lyapunov function is given as


(39) $$\begin{array}{l} V=\frac{1}{2}s_{i}^{2}. \end{array}$$

The corresponding derivative is given as


(40) $$\begin{array}{l} {\dot{V}}_{i}=s_{i}{\dot{s}}_{i},\\ \;=s_{i}(-{\tilde{W}}_{i}^{T}\varphi_{i}(x)-\eta_{i}sgn(s_{i})+d_{i}+\varepsilon_{i}),\\ \;\leq -|s_{i}|(\eta_{i}+{\tilde{W}}_{i}^{T}\varphi_{i}(x)-\kappa_{i}). \end{array}$$

Considering that the Gauss function φ_*i*_(*x*) and $-{\tilde{W}}_{i}^{T}\varphi_{i}\left(x\right)+\kappa_{i}$ are both bounded, when the gain η_*i*_ satisfies the condition that $\eta_{i}>-{\tilde{W}}_{i}^{T}\varphi_{i}\left(x\right)+\kappa_{i}$, there is


(41) $$\begin{array}{l} {\dot{V}}_{i}\leq -\gamma_{i}|s_{i}|=-\gamma_{i}\sqrt{V_{i}}, \end{array}$$

where $\gamma_{i}=\eta_{i}+{\tilde{W}}_{i}^{T}\varphi_{i}\left(x\right)-\kappa_{i}$. As long as the parameter η_*i*_ is reasonably designed, the convergence of the tracking error is ensured, and the sliding mode variable do converge to some neighborhood of zero. In addition, the size of the convergence neighborhood depends on the control parameter; that is, by properly design the control parameters, ideal synchronization control performance can be achieved.

***Remark 4:*** According to the relearning-based sliding mode control algorithm given above, if there is no dynamic pattern that is sufficiently similar to the unknown slave system in the pattern base χ, the on-line identification process for the unknown slave system is started. Different from the initial identification process, the initial weights of the neural network during the identification process for unfamiliar synchronization objects are taken from the constant weight of the dynamic system corresponding to the minimum recognition error rather than iterating from zero. Thus, the learned and stored dynamic information help reduce the on-line identification time. Additionally, the identified dynamic information of the slave system will be restored in the form of constant weights and can further utilized to new synchronization problems. This process will help enrich the empirical dynamics information of the pattern base to improve the accuracy and efficiency of the new synchronization tasks.

## 7. Simulation research

To verify the effectiveness of the control strategy proposed in this study, simulations of the master-slave FOHR system under unknown dynamic environment are conducted.

### 7.1. Identification of the unknown dynamics of the FOHR system

In this part, the identification of the FOHR system shown in Equation (6) under unknown dynamic environment is considered. For the convenience of presentation, the system state *x*, *y*, and *z* are denoted as *x*_1_, *x*_2_, and *x*_3_, respectively. The corresponding state vector $x={\left[x_{1},x_{2},x_{3}\right]}^{T}\in R^{3}$ of the FOHR model is available from measurement and the parameter $\mu ={\left[a,b,c,d,r,s_{0},q_{0}\right]}^{T}$ is taken as a constant vector and chosen as *a* = 1, *b* = 3, *c* = 1, *d* = 6, *r* = 0.013, *s*_0_ = 4, *q*_0_ = −1.56. As demonstrated in Section 3, by varying the fractional order parameter *q* and fixing all the other parameters unchanged, the FOHR system presents diverse non-linear behaviors. Moreover, the 0.98-order HR model can best describe the abundant non-linear dynamic characteristic of neurons. Thus, the 0.98-order HR system is considered for dynamic identification with the external excitation *I* being taken as the control parameter.

To verify the identification effects, four kinds of representative discharge models of the 0.98-order HR system with the parameters given above are chosen, that is, the slow-spiking model χ^1^ with *I* = 1.5, the period-3 bursting model χ^2^ with *I* = 2.5, the chaotic bursting model χ^3^ with *I* = 3.6 and the fast-spiking model χ^4^ with *I* = 4. The dynamic analysis about FOHR system have demonstrated that the corresponding state trajectories of the four dynamic models mentioned above possess regression properties. Thus, the DL algorithm is introduced for the unknown dynamic identification process.

According to the DL algorithm, the dynamical RBF network $\dot{\hat{x}}=-A\left(\hat{x}-x\right)+Ŵ\phi \left(x\right)$ is employed to identify the unknown system dynamics *f*_*i*_(*x*; μ)(*i* = 1, 2, 3) as shown in Equation (6). For the space limitation, the unknown dynamic *f*_3_(*x*; μ) = *r*(*s*_0_(*x*−*q*_0_)−*z*) is taken as an example to show the identification effects. The center of the neural network is evenly placed on [−2.1, 2.1] × [0.9, 5.1] and the widths are set as η_*i*_ = 0.3. The weights of the RBF networks are updated online according the equation ${\dot{Ŵ}}_{i}={\dot{\tilde{W}}}_{i}=-\Gamma_{i}\varphi_{i}\left(x\right){\tilde{x}}_{i}-\sigma_{i}\Gamma_{i}{Ŵ}_{i}$, within which the parameters are chosen as Γ_*i*_ = *diag*{2, 2, 2}, σ_*i*_ = 0.0001, *i* = 1, 2, 3 and *a*_3_ from $A={\left[a_{1},a_{2},a_{3}\right]}^{T}$ is set as *a*_3_ = 10. The initial condition of the dynamical system is set as ${\left[x_{1}\left(0\right),x_{2}\left(0\right),x_{3}\left(0\right)\right]}^{T}={\left[0.3,1,3\right]}^{T}$, ${\left[{\hat{x}}_{1}\left(0\right),{\hat{x}}_{2}\left(0\right),{\hat{x}}_{3}\left(0\right)\right]}^{T}={\left[0.2,0.3,0.0\right]}^{T}$, and the initial weights are Ŵ_*i*_(0) = 0.0.

**Figure 8:**
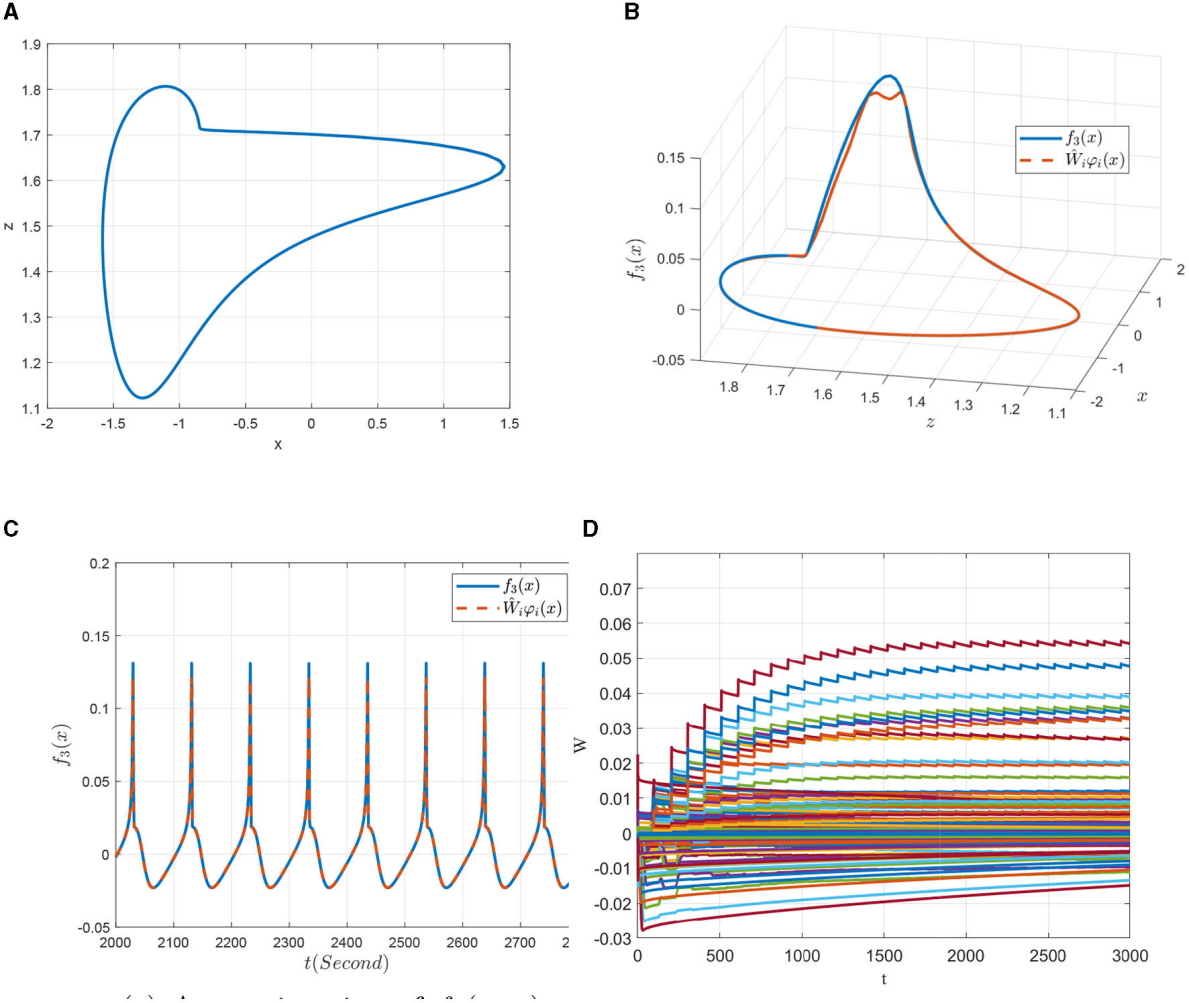
Non-linear dynamic identification of the 0.98-order HR system with *I* = 1.5 (χ^1^). **(A)** State trajectory on the *x* − *z* plane. **(B)** Approximation of the state trajectory. **(C)** Approximation of *f*_3_(*x*; μ). **(D)** Weight convergence.

First, the 0.98-order HR system with external excitation *I* = 1.5 denoted in a slow-spiking model as χ^1^ is to be identified. Figure 8A is the projection of the state trajectory of the slow-spiking model on the *x*−*z* plane. In Figures 8B, C, it is seen that the state trajectory can be accurately identified by using the DL algorithm. More importantly, in addition to the state tracking, the NN approximation of the system dynamics *f*_3_(*x*; μ) along the system trajectory is shown in Figure 8C. The convergence of the weights of the RBF neural network is further obtained from the Figure 8D. That is, by introducing the DL algorithm, the unknown dynamic information *f*_3_(*x*; μ) of the FOHR model is locally accurately approximated by Ŵ_*i*_φ_*i*_(*x*), and the identified non-linear dynamic information can be further stored in the constant weights of networks given as ${\bar{W}}_{i}\varphi_{i}\left(x\right)$.

**Figure 9:**
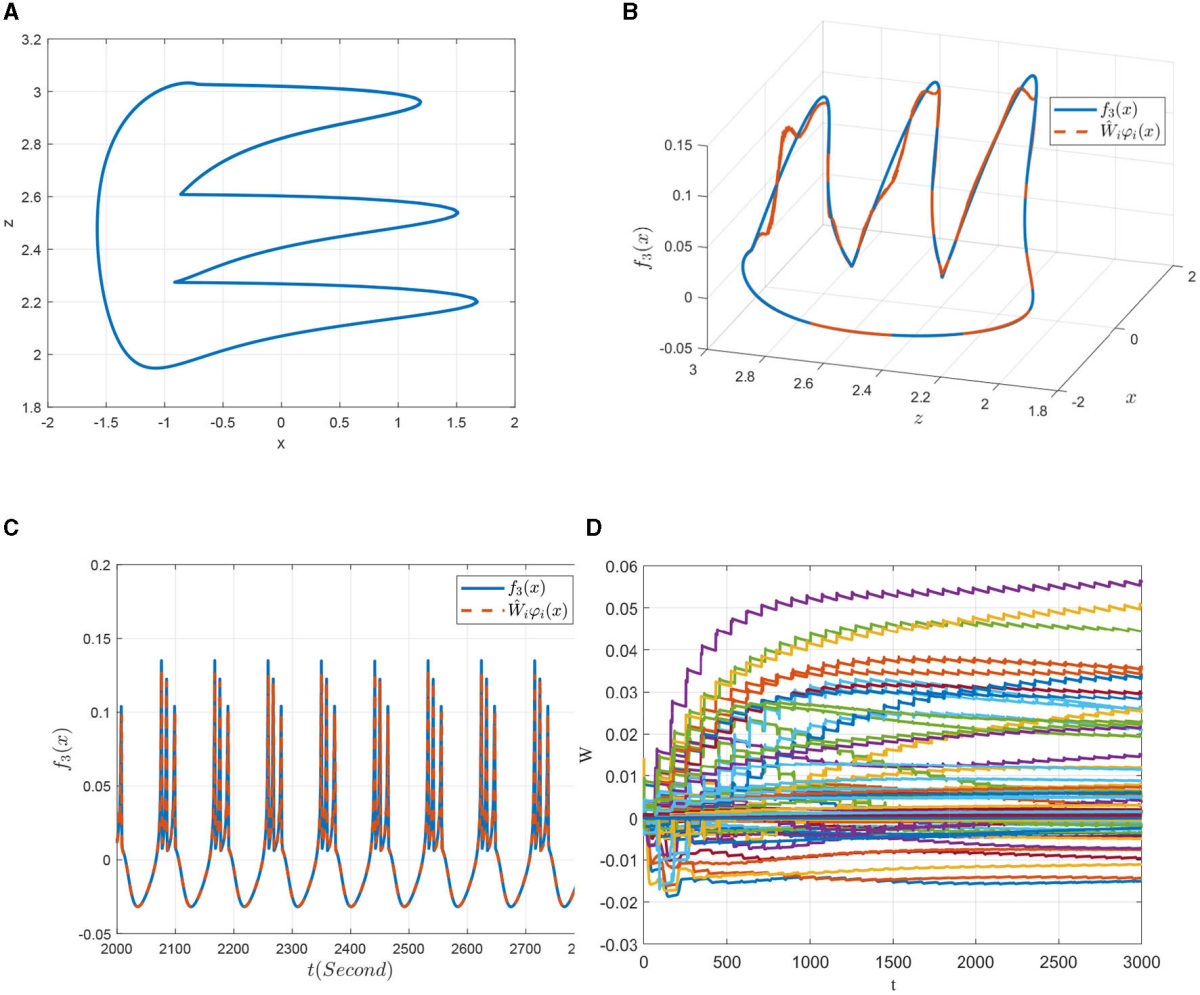
Non-linear dynamic identification of the 0.98-order HR system with *I* = 2.5 (χ^3^). **(A)** State trajectory on the *x* − *z* plane. **(B)** Approximation of the state trajectory. **(C)** Approximation of *f*_3_(*x*; μ). **(D)** Weight convergence.

Second, similar results are obtained for the identification of the non-linear dynamics of the 0.98-order HR system with *I* = 2.5 that exhibiting a period-3 bursting model denoted as χ^2^. It can be seen from the Figure 9A that the non-linear dynamics of the period-3 bursting model are richer than that of the dynamics presented in Figure 8A. Even though, ideal approximation effects of both the system state and the unknown system function are obtained as demonstrated in Figures 9B, C. The parameters of the corresponding RBF networks also converge to an ideal value, which can be seen from Figure 9D.

**Figure 10:**
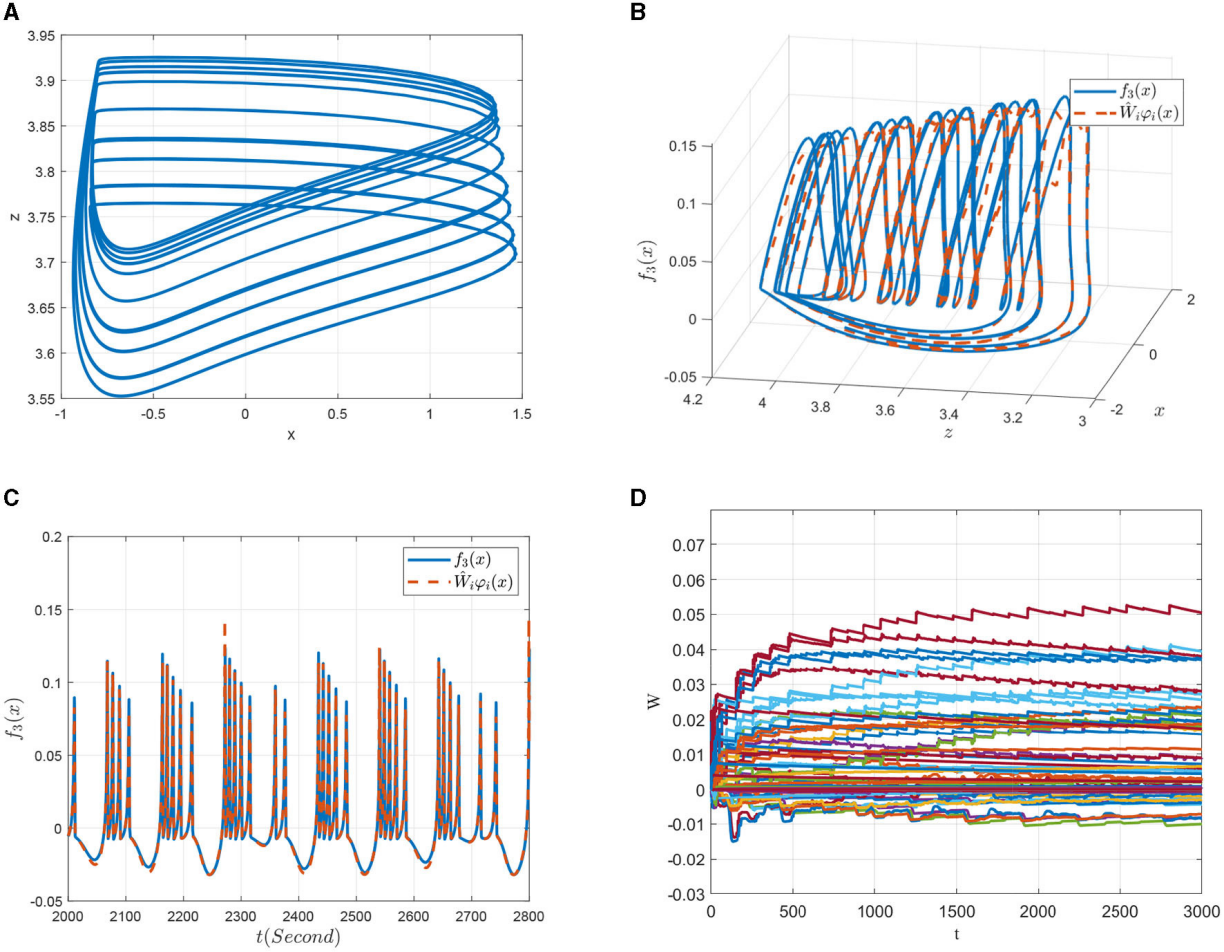
Non-linear dynamic identification of the 0.98-order HR system with *I* = 3.6 (χ^3^). **(A)** State trajectory on the *x* − *z* plane. **(B)** Approximation of the state trajectory. **(C)** Approximation of *f*_3_(*x*; μ). **(D)** Weight convergence.

Third, consider the identification of the dynamics of model χ^3^ with *I* = 3.6, as shown in Figure 10. The system state given in Figure 10A presents a complex state of chaos, which contains more dynamic information of the FOHR system. By properly designing the identification parameters, locally accurate NN approximations of the system state as well as the unknown system dynamics are achieved along the system trajectory, which can be seen from Figures 10B, C. In addition, it is noticed from the Figure 10D that more neurons are involved and activated in the identification of the chaotic bursting model χ^3^. Moreover, the oscillation of the NN weights during the convergence process is so obvious that more time is needed for it converge to the ideal values.

**Figure 11:**
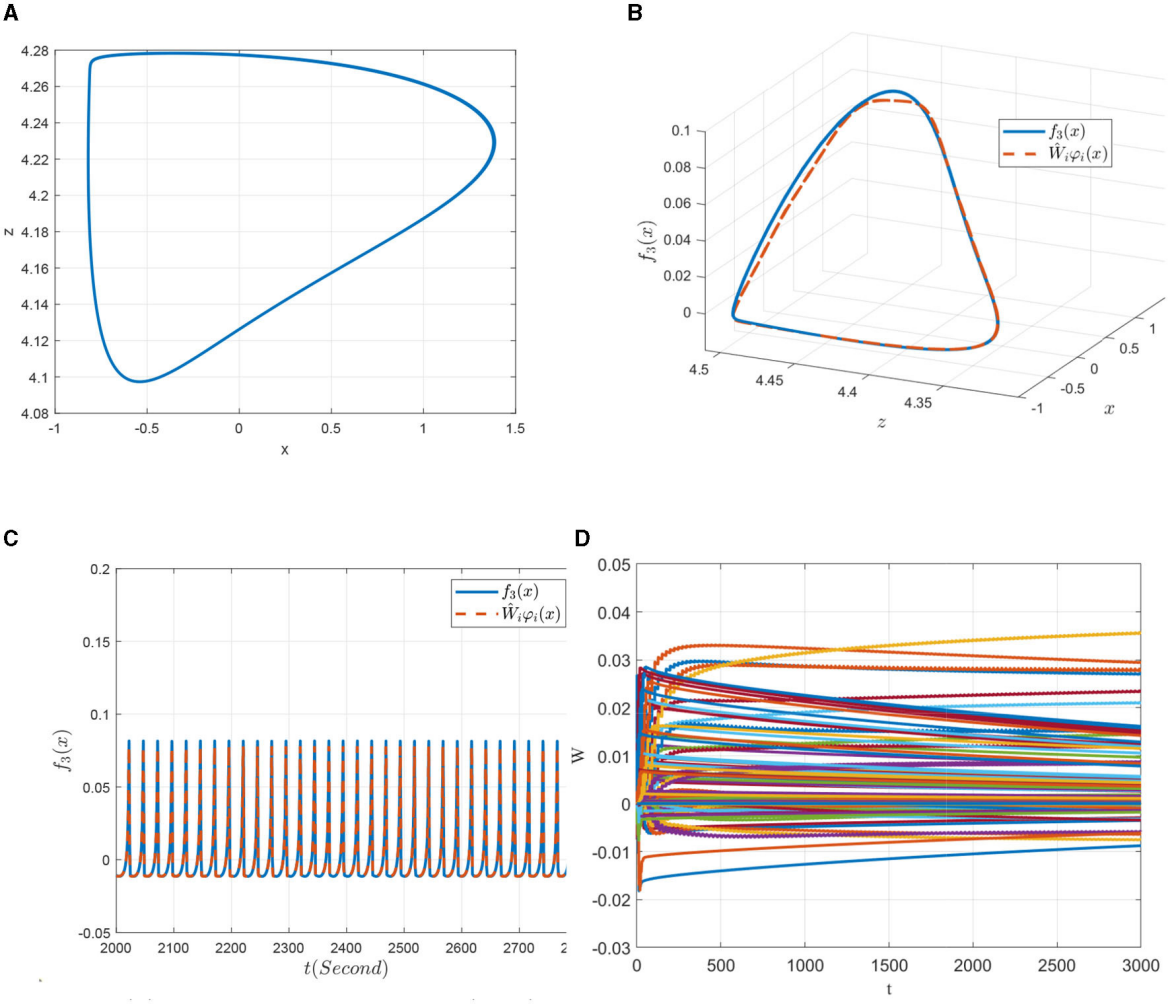
Non-linear dynamic identification of the 0.98-order HR system with *I* = 4 (χ^4^). **(A)** State trajectory on the *x* − *z* plane. **(B)** Approximation of the state trajectory. **(C)** Approximation of *f*_3_(*x*; μ). **(D)** Weight convergence.

Finally, a further increase in the external excitation *I* to 4 (denoted as model χ^4^), the system returns back to a simple discharge state. As can be seen from Figure 11A, the state trajectory of model χ^4^ is a typical period-1 behavior, but the discharge rhythm is faster compared to that of the model χ^1^ shown in Figure 8A. As for its identification simulations demonstrated in Figures 11B–D, it is shown that it achieves better state and dynamic tracking effects, and the parameter convergence process is much smooth and faster.

### 7.2. Model-based sliding-mode control of the FOHR system

Based on the acquisition and storage of the unknown dynamic information of the FOHR system, the rapid recognition of the FOHR model is demonstrated in this part. The dynamic models χ^1, 2, 3, 4^ mentioned above are taken as the training patterns. The testing patterns are generated from the FOHR system presented in Equation (7), with *I* = 1.43 denoted as χ^5^, *I* = 2.3 denoted as χ^6^, *I* = 3.4 denoted as χ^7^, and *I* = 4.2 denoted as χ^8^. The other parameters are set as the same to the training patterns, that is, *q* = 0.98, *a* = 1, *b* = 3, *c* = 1, *d* = 6, *r* = 0.013, *s* = 4, and *q*_0_ = −1.56. For the recognition process, the dynamic NN network system is introduced, that is,


(42) $$\begin{array}{l} {\dot{\tilde{x}}}_{i}=-{\bar{b}}_{i}{\tilde{x}}_{i}+\left({\bar{W}}_{i}^{kT}\varphi_{i}\left(x\right)-f_{i}^{\prime }\left(x;\mu^{\prime }\right)\right),i=1,\cdots \;,n \end{array}$$

for which, the initial states is given as ${\left[x_{0},y_{0},z_{0}\right]}^{T}={\left[0.3,1,3\right]}^{T}$ and ${\left[{\tilde{x}}_{0},{ỹ}_{0},{\tilde{z}}_{0}\right]}^{T}={\left[0,0,0\right]}^{T}$.

Based on the obtained dynamic pattern database χ, which contains the learned system dynamics as experience of the slaw peak regular spiking model, period-doubling, period-3, period-4 bursting model, and chaotic bursting model, the simulation of the learning-based sliding-mode control of the master-slave neural system is discussed in this part. The corresponding parameters are given as η_*i*_ = 1, *c*_*i*_ = 1, Γ_*i*_ = 2, and σ_*i*_ = 0.01, (*i* = 1, ⋯ , *n*). The external disturbances are set as *d*_1_(*t*) = 0.6 + 0.2*cos*(*t*), *d*_2_(*t*) = 0.0, *d*_3_(*t*) = 0.01 + 0.05*sin*(*t*), and the other parameters of the master-slave system are given as the same as shown in the previous section. The external stimulus current of the master system is set as *I* = 1.5, while for the slave system, the external stimulus current is set as *I* = 3.8. The other parameters are designed as *q* = 0.98, *a* = 1, *b* = 3, *c* = 1, *d* = 6, *r* = 0.013, *s* = 4, and *q*_0_ = −1.56, the initial state of the master-slave system is given as ${\left[x_{0},y_{0},z_{0}\right]}^{T}={\left[0.313\right]}^{T}$, and the control will be added at *t* = 300*ms*.

**Figure 12:**
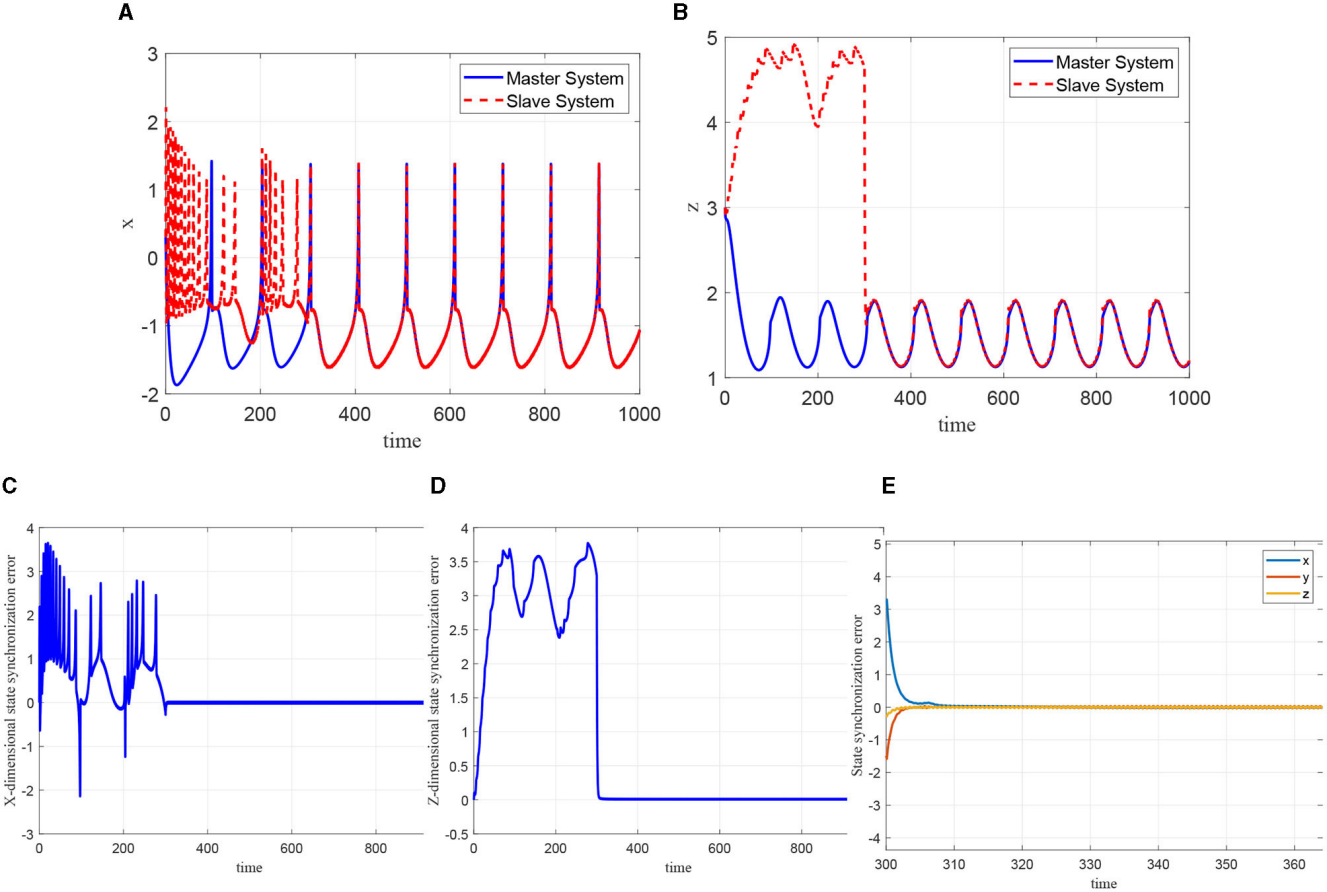
Synchronization of the master-slave neuron system. **(A)** Synchronization of *x*. **(B)** Synchronization of *z*. **(C)** Synchronization error of *x*. **(D)** Synchronization error of *z*. **(E)** Synchronization error of *x, y, z*.

As can be seen from the Figures 12A, B, when the control quantity is added to the slave system at *t* = 300*ms*, the state of the master-slave neurons can quickly reach consistency, and the selected NN controller achieves good synchronization to the master neuron system. Moreover, the synchronization error demonstrated in Figures 12B–D shows that the FOHR master-slave neuronal system achieves fast synchronization performance.

**Figure 13:**
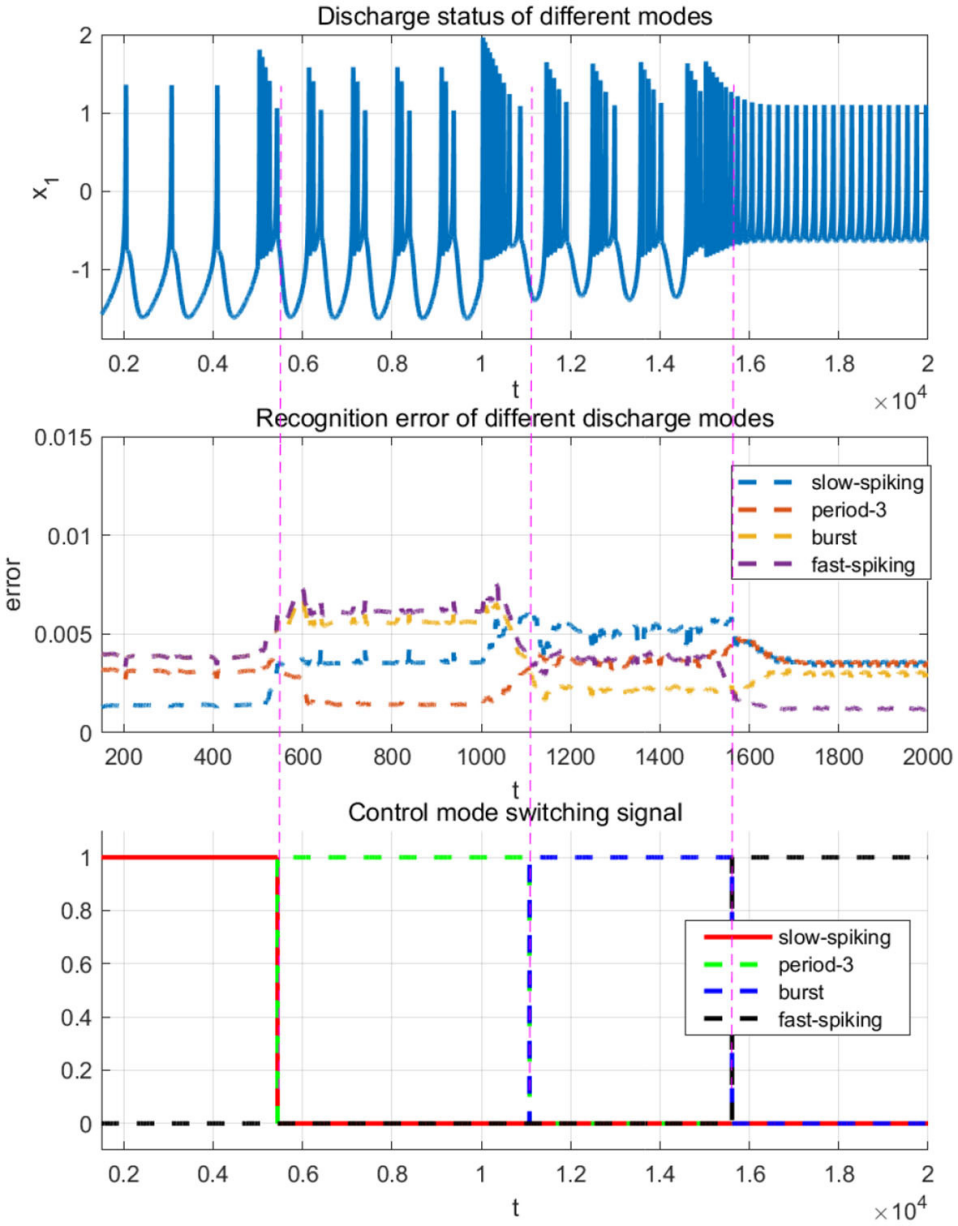
Switching control based on recognition error.

Since the external excitation of the master and slave system are set as *I* = 1.5 and *I* = 3.8, respectively, it means that the master system is in slow-spiking state and the slave system is in a state of rapid-peak spiking, as described in the identification phase. For accurate synchronization effect, the rapid-peak spiking model shown be recalled from the pattern base χ^4^, which can be validated from the Figure 13.

**Figure 14:**
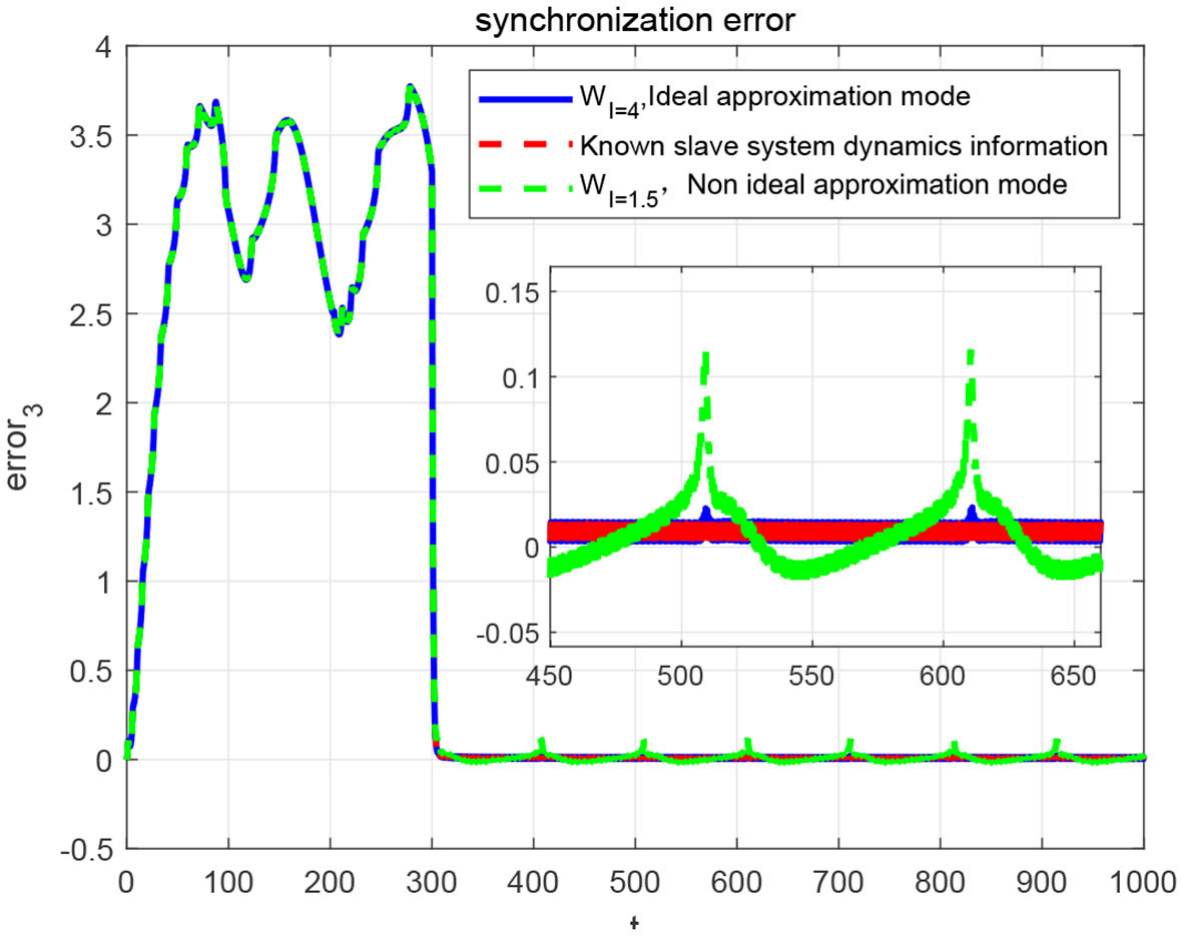
Synchronization error of master-slave neuron system with different modes.

In addition, through the simulation comparison by recalling the rapid-peak spiking model, the ideal known dynamic model corresponding to the slave system and the slow-spiking model, respectively, the synchronization errors are shown in Figure 14. It demonstrate that in terms of convergence speed, accuracy, and buffeting size, the more accurate the dynamic model is selected, the better the synchronization effect will be. It further indicates that the performance of the sliding-mode control algorithm is highly related to the dynamic information accuracy of the invoked dynamic models.

### 7.3. Relearning-based sliding-mode control of the FOHR system

**Figure 15:**
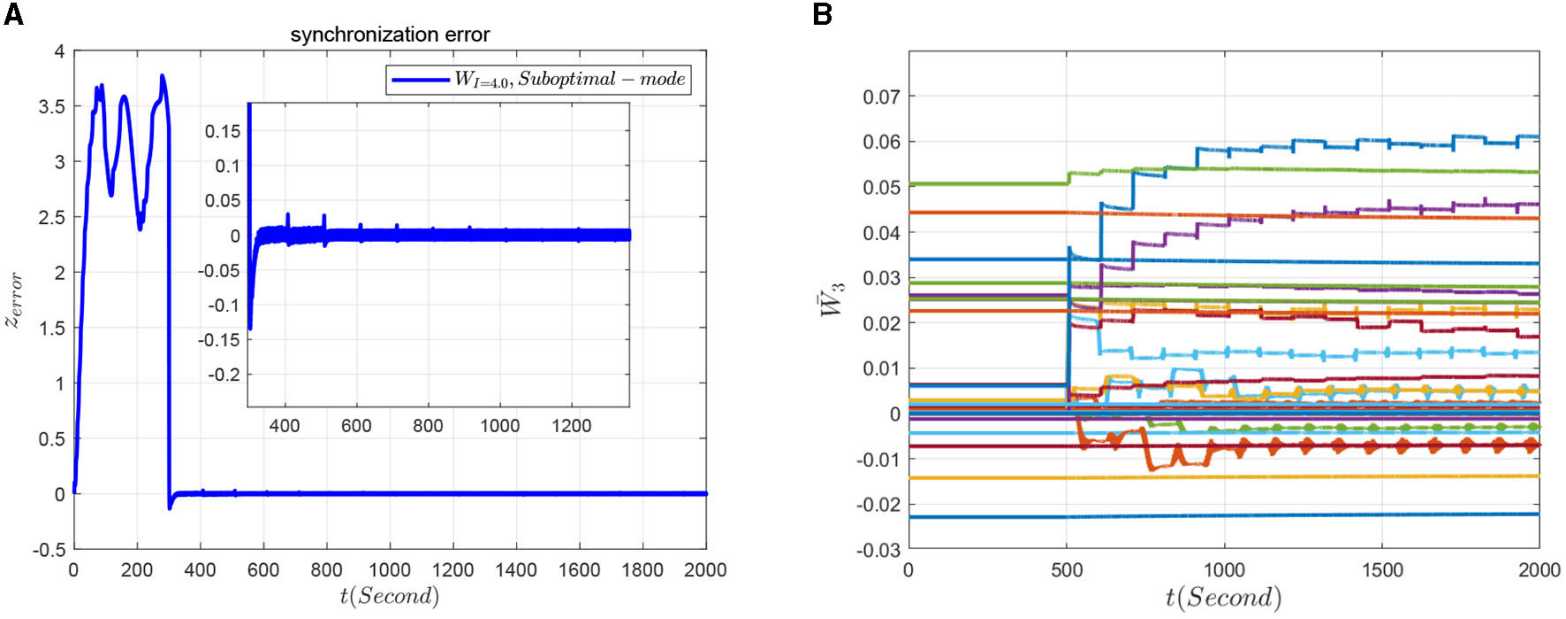
Synchronization effect of the master-slave neuron system based on relearning control. **(A)** Synchronization error. **(B)** Convergence of the NN weight.

To verify the effectiveness of the relearning-based sliding-mode control performance to the master-slave neuronal system, the third dimension dynamics of the neuron system is taken as an example, and the sliding-mode controller is set as


(43) $$\begin{array}{l} \begin{array}{c} \mu_{3}=-\eta_{3}sgn\left(s_{3}\right)-c_{3}e_{3}+f_{m,3}\left(x\right)-{Ŵ}_{3}^{T}\varphi_{3}\left(x\right),\\ \mu_{3}\left(0\right)=-\eta_{i}sgn\left(s_{3}\right)-c_{i}e_{3}+f_{m,3}\left(x\right)-{\bar{W}}_{3}^{K_{0}T}\varphi_{3}\left(x\right), \end{array} \end{array}$$

in which the initial NN weight is set as ${Ŵ}_{3}\left(0\right)={\bar{W}}_{3}$, with ${\bar{W}}_{3}$ being the constant NN weight. During the control process, the model-based sliding mode controller is added to the system at 300 ms and at 500 ms switch to the relearning-based sliding mode controller. The synchronous response of the system can be seen from Figure 15A. Furthermore, it can be seen from Figure 15A, when the system switch to the relearning-based sliding mode control policy, the synchronization error is getting smaller because of more accurate identification of the dynamics of slave system, and the cusp error is obviously improved. In addition, the NN weight of the relearning process can convergence to ideal values as shown in Figure 15B.

## 8. Conclusion

Aiming at the problem of abnormal synchronization of fractional-order Hindmarsh-Rose (FOHR) neuronal system in unknown dynamic environment, the identification, rapid recognition, and synchronization control of the unknown dynamic FOHR system is discussed in this study. For accurate synchronization of the FOHR neuronal system, the unknown dynamic information has been identified by using the deterministic leaning theory. Based on the achieved system dynamics, the unknown different dynamic patterns generated from the FOHR system can be rapidly recognized without relearning process. In addition, the achieved dynamic information has been applied to the sliding mode controller, resulting in more accurate and efficient synchronization performance of the master-slaver neuronal system. From system identification to pattern construction, then to model-based and relearning-based sliding mode control, this study emphasizes the whole linkage process, which kindly displays the human experience of learning and application of unknown knowledge, which is the essence of intelligent learning and intelligent control.

## Data availability statement

The original contributions presented in the study are included in the article/supplementary material, further inquiries can be directed to the corresponding author.

## Author contributions

DC, JL, and CY contributed to the methodology, concept, and design of the study. DC, JL, and JH performed the experiments and simulation analysis. JL, JH, and WZ prepared the draft manuscript. All authors participated manuscript organization and approved the submitted version.
